# Ensemble Analyzer: An Open-Source Python Framework for Automated Conformer Ensemble Refinement

**DOI:** 10.1021/acs.jcim.6c00273

**Published:** 2026-04-21

**Authors:** Andrea Pellegrini, Paolo Righi, Andrea Mazzanti, Michele Mancinelli

**Affiliations:** Department of Industrial Chemistry “Toso Montanari”, Alma Mater Studiorum University of Bologna, Via P. Gobetti 85, 40129 Bologna, Italy

## Abstract

Accurate prediction of molecular properties requires the refinement of the full conformational ensemble at high levels of theory, which remains computationally demanding. Existing workflows typically rely on *ad-hoc* scripts and manual intervention. We present Ensemble Analyzer (EnAn), an open-source Python framework that automates this workflow. EnAn’s modular and extensible architecture integrates seamlessly with widely known calculators and allows automated generation and comparison of electronic and vibronic spectra. EnAn effectively manages the reproducible exploration of conformational spaces.

## Graphical abstract

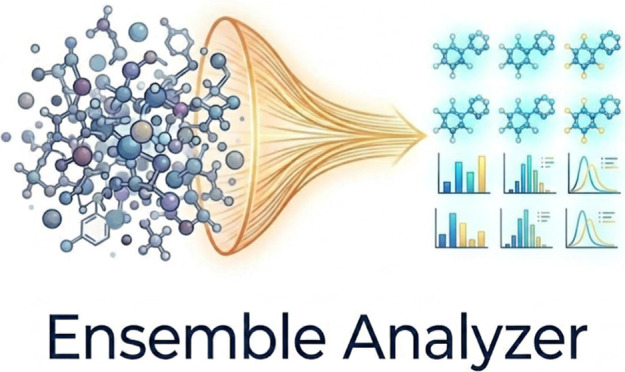

## 1 Introduction

Conformational sampling has recently been established as a cornerstone of modern computational organic chemistry.^1–5^ The conformational landscape of flexible molecules profoundly influences their physical and chemical properties, and even minimal structure variations might dramatically alter the reactivity, spectroscopic signatures, and molecular assembly. Consequently, the accurate prediction of computed energetics and spectra depends on Boltzmann-weighted contributions of the widest representative ensemble possible. Neglecting this critical step often leads to significant discrepancies between the computed and experimental results.

Over the past five years, the conformational search field has undergone a profound transformation, pushed by the emergence of open-source tools such as RDKit,^6^ CREST,^7,8^ PyConSolv,^9^ and most recently GOAT^10^ (integrated within ORCA^11,12^ ). These software packages have democratized chemical space exploration, enabling a rapid generation of extensive ensembles, often comprising hundreds to thousands of conformers, at minimal computational cost. This efficiency is achieved through relatively inexpensive methods, ranging from classical molecular dynamics to meta-dynamics based on semiempirical extended tight-binding (xTB^13,14^ ) Hamiltonians. Notably, xTB methods are increasingly becoming among the most widely used for conformational sampling, offering a favorable balance between accuracy and computational speed.

Despite these advances, the significant bottleneck remains in the downstream processing: the refinement of all the conformers at higher levels of theory, required for improving accuracy, remains computationally prohibitive for large ensembles. Conventional protocols have often focused on identifying the single global minimum^15^ of the ensemble and carrying it—perhaps few others—over to further higher-level calculations. However, this reductionist approach is inadequate, especially for systems with flat potential energy surfaces, where multiple thermally accessible minima contribute significantly to ensemble-averaged properties and cannot be reliably approximated by a single representative structure.

Bridging the gap between raw ensemble generation and rigorous quantum mechanical (QM) characterization is challenging. Current workflows^16–19^ present a rigid infrastructure and/or require the creation of custom scripts to filter structures, manage input/output across different QM engines, and parse results. This technical barrier not only limits the reproducibility of studies but also confines advanced ensemble analysis exclusively to users with strong scripting skills, thus hampering broader adoption of best practices in conformational sampling.

To overcome these limitations, here, we introduce Ensemble Analyzer (EnAn), a flexible and automated software tool designed to streamline the refinement of conformational ensembles. Unlike command-line utilities, EnAn provides an intuitive, user-friendly interface that is accessible to researchers without programming expertise. The software automates the entire workflow: from job submission and output parsing to ensemble pruning, duplicate removal, and dimensionality reduction through Principal Component Analysis (PCA)^20^-guided clustering that preserves structural information and diversity. EnAn further supports the automatic generation, optimization, and comparison of electronic and vibronic spectra, enabling rapid visualization and interpretation of results (see the Supporting Information for a more detailed comparison between EnAn and some of existing tools). By minimizing manual data handling and standardizing workflows, EnAn promotes efficient and reproducible exploration of the complex conformational space.

## 2 Workflow

EnAn has been developed to automate repetitive quantum chemical calculations over externally generated conformational ensembles. The software architecture follows a modular design based on the Atomic Simulation Environment^21^ framework, enabling seamless integration of multiple quantum chemistry engines. The current version implementation supports the implementation of ORCA (version 5.0 and above) and Gaussian16,^22^ with the modular structure allowing future extensions to additional QM engines.

The complete workflow (illustrated schematically in Figure 1) requires only two input files: (i) the ensemble geometries in standard XYZ format and (ii) a protocol file defining the computational steps. The protocol is formatted as a JSON file that specifies the sequential calculation steps, each with customizable parameters including level of theory, solvent model, optimization and frequency switches, and pruning thresholds. To streamline protocol creation, an interactive script is provided, guiding users through the setup with three complexity levels: Basic, Intermediate, and Advanced.

**Figure 1:**
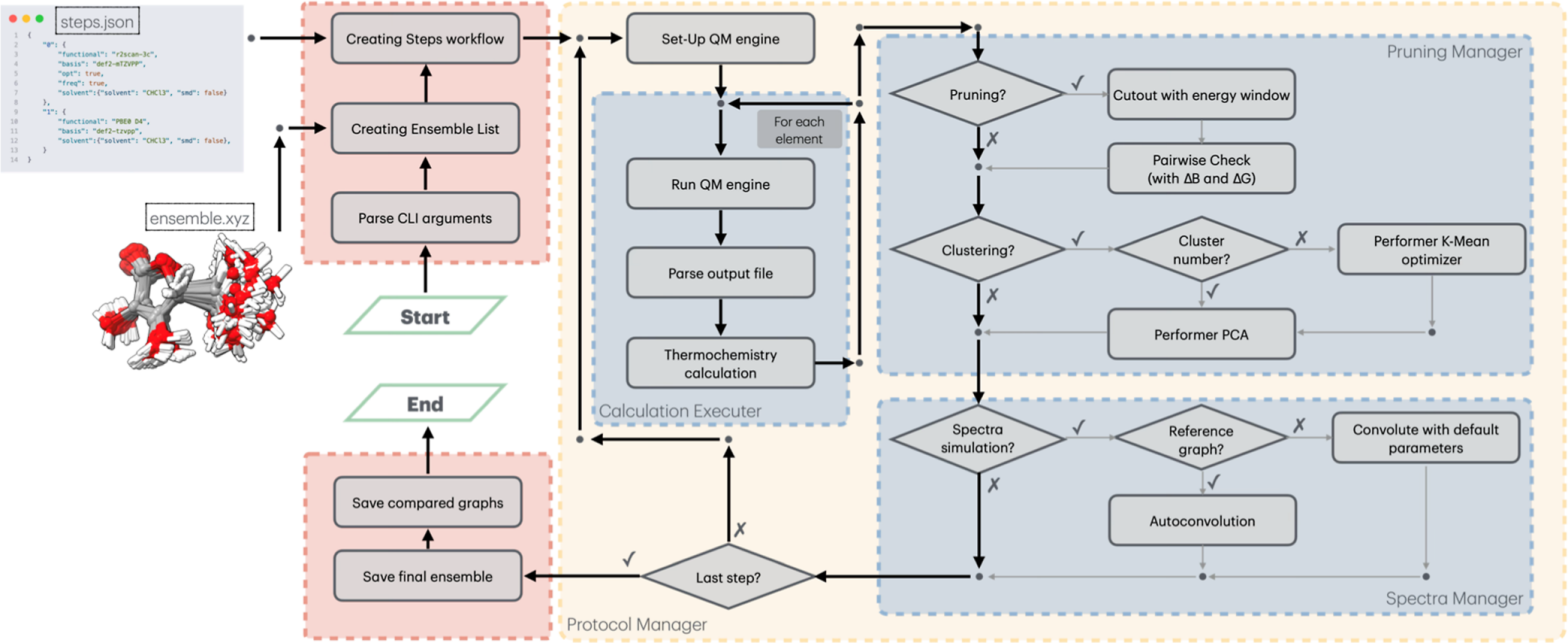
Schematic workflow of Ensemble Analyzer (EnAn).

EnAn implements Boltzmann weighting of conformers based on Gibbs free energies evaluated using the quasi-Rigid Rotor Harmonic Oscillator (qRRHO) approximation with Grimme’s damping scheme for low-frequency modes.^23^ To ensure consistency across different QM engines, EnAn internally recalculates partition functions and thermodynamic contributions by extracting raw data needed for these calculations from output files and applying Grimme’s qRRHO formulas uniformly, regardless of the backend computational engine. Relative populations are automatically computed at the specified temperature and used to weight individual conformer contributions to ensemble-averaged properties and spectra.

To efficiently manage the ensemble size and remove redundant structures, EnAn implements a computationally lightweight dual-filter pruning strategy. Unlike traditional methods that rely primarily on Cartesian Root-Mean-Square Deviation (RMSD) alignments, which require expensive geometry alignments of structures, EnAn uses the scalar norm of the rotational constant vector (B) and the energy (Gibbs energy G, when available; otherwise, electronic energy E is used) as the primary filtering metrics. Two conformers are considered identical—and the one with higher relative energy discarding—if they simultaneously satisfy both an energetic (|*E*_*i*_ – *E*_*j*_| < *thrG*) and a geometric (|*B*_*i*_ – *B*_*j*_| < *thrB*) equivalence condition. This approach conceptually parallels the fast-filtering heuristics employed by conformer generators, such as CREST, but it is strictly implemented as a pairwise scalar comparison to bypass structural alignments entirely during the refinement steps. The pruning thresholds (thrG, thrB, and thr*G*_max_) are dynamically selected based on the nature of the computational step. A detailed description of the filtering algorithm, along with the standard default parameters used for all job types, is provided in Section 1.1 of the Supporting Information.

Optional PCA-based clustering can be activated on demand through the protocol settings. This PCA analysis operates on the eigenvalues of atomic distance matrices computed from Cartesian coordinates with an option to include or exclude hydrogen atoms. This flexibility allows users to focus on heavy-atom skeletal differences or include all atoms for a complete conformational description. When clustering is enabled, conformers are grouped using K-means clustering, with either automatic determination of optimal cluster number via silhouette score maximization or user-defined cluster count. For each cluster, the representative structure is automatically selected as the lowest-energy conformer assigned to that cluster centroid. Initial cluster centroids are randomly seeded and then iteratively optimized through the K-means algorithm to maximize the silhouette score, ensuring both energetic favorability and conformational diversity in the reduced ensemble.

For spectroscopic applications, EnAn automatically generates ensemble-averaged IR, vibrational circular dichroism (VCD), UV, and electronic circular dichroism (ECD) spectra from the Boltzmann-weighted conformer ensemble when frequency calculations or electronic state energies are requested. Individual conformer spectra are first convoluted using appropriate line shape (LS) functions (Lorentzian for IR/VCD and Gaussian for UV/ECD) with theory-dependent broadening parameters, then combined according to their population weights. When experimental reference spectra are provided (as simple two-column XY data files), EnAn implements an automated optimization routine to determine the optimal spectral alignment parameters. The algorithm simultaneously optimizes both spectral shift (multiplicative scaling for IR/VCD wavenumbers or additive shift for UV/ECD energies) and Full-Width at Half-Maximum (FWHM) of the convolution function by minimizing the RMSD between calculated and experimental spectra within the recorded experimental window. This automated fitting procedure, performed using the L-BFGS-B^24^ optimization method with user-definable boundaries, eliminates manual spectral manipulation and provides quantitative similarity metrics between theory and experiment. Optimized spectra, along with fitting parameters and similarity scores, are saved for each protocol step, enabling a systematic evaluation of how the level of theory affects spectral predictions.

## 3 Usage

### 3.1 Ensemble Refinement

Our first case study demonstrates a multilevel automated refinement workflow, illustrating the typical process for ensemble refinement and the subsequent reduction of conformers. Using CREST at the GFN2-xTB level, we have generated the ensembles for several medium to large, highly flexible molecules: Diethyl phthalate, Guaiol, Penicillin V, Permethrin, Rivaroxaban, Sitagliptin, Tamiflu, and Ascorbic acid. Each ensemble was then subjected to a three-step refinement protocol: (i) single point at the g-xTB^25^ level, (ii) geometry optimization at the r^2^SCAN-3c^26^ level, and (iii) a single-point electronic energy computation at the ωB97X-D4rev^27,28^ /def2-QZVPP^29^ level. All calculations were performed using ORCA 6.1.0. Default thresholds were applied, consistent with CREST parameters for identifying new conformers, and the maximum energy window was chosen (3.50 kcal/mol after optimization and frequency calculation) to include all conformers with significant Boltzmann populations. The initial single-point energy window was expanded to account for high-energy structures that might undergo significant stabilization during following geometry optimizations. Specifically, single-point calculations employ a wider energy threshold (Δ*G*_max_ = 6 kcal/mol) compared to optimization steps, allowing potentially relevant conformers to be carried forward before refinement. All threshold parameters can be manually adjusted in the protocol definition file by the user. Detailed default energy windows for each job type are provided in the Supporting Information (Table S2). The results of the refinement process are summarized in Table 1.

**Table 1:**
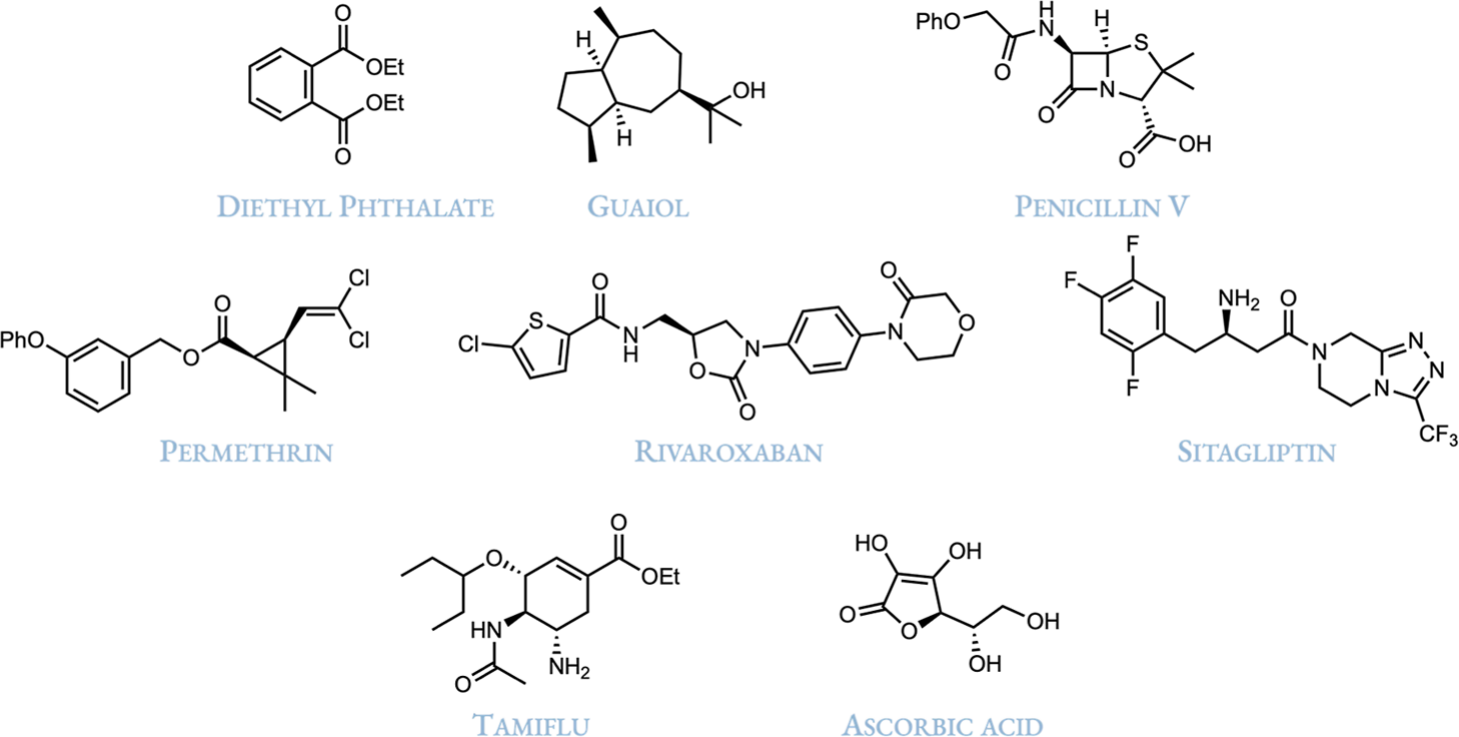
Refinement Process of Highly Flexible Molecules^a^

	starting ensemble	protocol 1	protocol 2	protocol 3	final retention rate
Diethylphthalate	156	151	35	35	22%
Guaiol	118	109	34	34	29%
Penicillin V	136	71	12	12	9%
Permethrin	304	304	101	101	33%
Rivaroxaban	110	110	30	30	27%
Sitagliptin	428	343	142	142	33%
Tamiflu	836	527	100	99	12%
Ascorbic acid	134	68	45	45	33%

a The number represents the number of conformers active after each protocol. Protocol 1: single point at the g-xTB level, Protocol 2: optimization and frequency calculation at r^2^SCAN-3c, and Protocol 3: single-point calculation at ωB97X-D4rev/def2-QZVPP.

### 3.2 Principal Component Analysis (PCA) Clustering

As illustrated in the previous example, conformational ensembles can become exceedingly large, making even single-point calculations on all conformers computationally demanding. To mitigate this computational cost, geometry clustering provides an effective strategy to reduce the number of conformers while preserving an effective description of the overall conformational space.

To evaluate the impact of clustering discretization on ensemble-derived properties, three flexible molecules were selected as representative case studies: Vancomycin, Paclitaxel, and Prostaglandin PGF_2α_ (see the Supporting Information Figure S2). Initial ensembles were generated using CREST, and the conformer energies were subsequently re-evaluated at the g-xTB level directly within EnAn. Each ensemble was then clustered while the number of resulting clusters. For each new clustered ensemble, the Boltzmann-weighted average energy was calculated and compared with that obtained from the full ensemble. The resulting trend, shown in Figure 2, reports the variation of the energy deviation (Δ*E*_av_, as defined in eqs 1–3) as a function of the percentage of conformers retained—thus illustrating the energy loss introduced by the progressive reduction in the number of clusters.

1 $$E_{av}=\sum_{i}^{N}p_{i}E_{i}$$

2 $$p_{i}=\frac{g_{i}\exp\left\{-\frac{\Delta E_{i}}{RT}\right\}}{\sum_{j}g_{j}\exp\left\{-\frac{\Delta E_{j}}{RT}\right\}}$$

3 $$\Delta E_{av}=E_{av,TOT}-E_{av,clustered}$$

**Figure 2:**
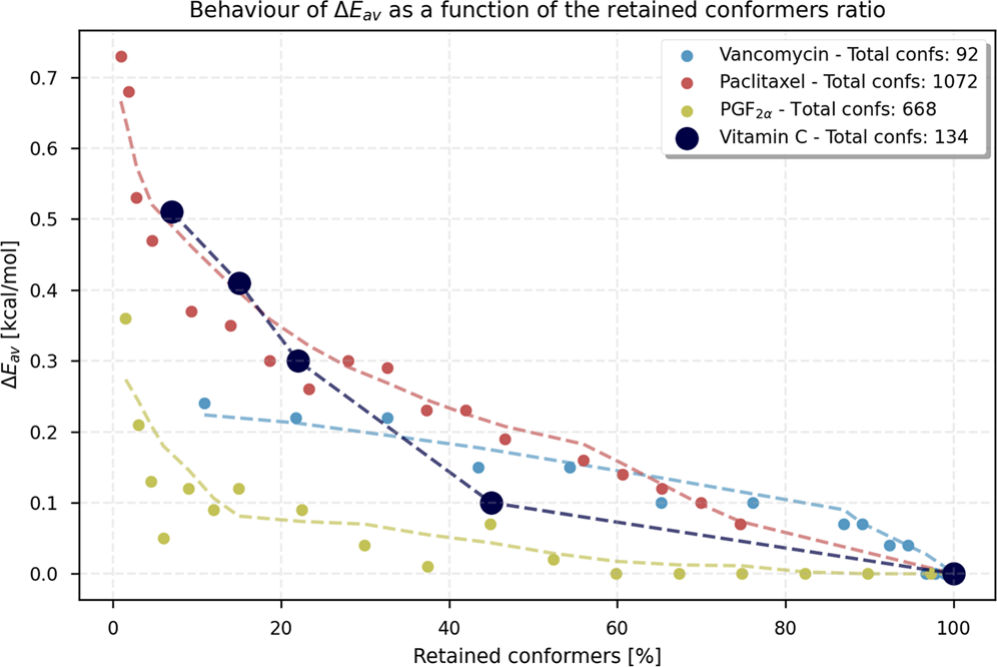
Variation of Δ*E*_av_ loss as a function of the conformer’s retention.

Ascorbic acid was also included; in this case, the values plotted were computed using Gibbs free energy, with electronic energy evaluated at the ωB97X-D4rev/def2-QZVPP level and the thermal correction calculated at the r^2^SCAN-3c level.

As expected, reducing the number of conformers decreases the accuracy, as reflected by the increase in Δ*E*_av_. Conversely, fewer conformers significantly reduce the wall time required for optimization and frequency calculation (Supporting Information Figure S4 shows how the number of clusters affects the accuracy and wall time of the computation). Therefore, clustering the ensemble represents an excellent strategy for reducing the dimensionality of the conformational space and, consequently, the computational cost. However, it must be applied judiciously to balance efficiency and accuracy. To identify the optimal clustering granularity, EnAn employs an automated optimization strategy. The algorithm systematically evaluates all integer values of n (number of clusters) between 10% and 80% of the ensemble size, with a lower bound of two clusters. For each n value, K-means clustering is performed, and the corresponding silhouette score is calculated. The configuration yielding the highest silhouette score is selected as the optimal clustering solution.

### 3.3 Spectra Simulation—Electronic and Vibrational Spectra

EnAn has been designed to manage also the most time-consuming aspects of multiconformer spectra simulation. EnAn can generate the electronic and vibrational spectra for all active conformers, weighting each contribution according to the Boltzmann population. Each output obtained from the QM engine is scaled by the conformer’s population and then convoluted with the appropriate LS for the corresponding spectrum. Users can customize the default convolution parameters for both LSs separately via the CLI flag “fwhm-vibro” and “fwhm-electro”, respectively. In the code, these LSs are implemented as shown in eqs 4 and 5

4 $$L\left(\omega_{i},\gamma \right)=\sum_{i}I_{i}\frac{\gamma_{i}^{2}}{\gamma^{2}+4{\left(X-\omega_{i}\Delta \right)}^{2}}$$

5 $$G\left(\omega_{i},\sigma \right)=\sum_{i}I_{i}\frac{1}{\sigma \sqrt{2\pi }}\exp\left[-\frac{1}{2}{\left(\frac{X-\left(\omega_{i}+\Delta \right)}{\sigma }\right)}^{2}\right]$$

where *L*(ω_*i*_,γ) denotes the Lorentzian convolution function, *I*_*i*_ is the intensity of the impulse, γ is the FWHM, and *X* is the entire frequency range considered for the convolution; *G*(ω_*i*_,σ) denotes the Gaussian convolution function, where σ is the broadening parameter calculated as $\sigma =\frac{\gamma }{\left(2\sqrt{2\ln\left(2\right)}\right)}$ . In both equations, Δ is the spectral shift applied to align the computed spectrum with the experimentally recorded spectrum.

If experimental spectra are present in the same folder, then the autoconvolute function is triggered. This routine optimizes the convolution parameters to achieve the best overlap between computed and experimental spectra using a bounded numerical minimization (via the minimize function from the scipy.optimize module^30^). The objective is to reduce the RMSD (eq 6) between the two spectra, yielding a similarity index^31,32^ as defined in eq 7, where max represents the maximum value of RMSD possible (1 for the achiral spectrum and 2 for the chiral ones). The weighting function *w*(*x*) focuses the action of the optimizer and is defined in eq 8.

6 $$RMSD=\frac{\sum_{i}{\left(y_{i}-y_{\exp}\right)}^{2}w\left(x_{i}\right)}{\sum_{i}w\left(x_{i}\right)}$$

7 $$S=1-\frac{RMSD}{\max}$$

8 $$w\left(x\right)=\{\begin{array}{l} 0\;\text{if}\;x<x_{\exp}^{\min}\text{or}x>x_{\exp}^{\max}\\ \max\left(0.15,\frac{g\left(x_{\mathrm{int}}^{\min},\sigma_{1}\right)}{\max\left(g\left(\min x_{\mathrm{int}},\sigma_{1}\right)\right)}\right)\;\text{if}\;x_{\exp}^{\min}<x<x_{\mathrm{int}}^{\min}\\ \begin{array}{l} \max\left(0.15,\frac{g\left(x_{\mathrm{int}}^{\max},\sigma_{2}\right)}{\max\left(g\left(\max x_{\mathrm{int}},\sigma_{2}\right)\right)}\right)\;\text{if}\;x_{\mathrm{int}}^{\max}<x<x_{\exp}^{\max}\\ 1\;\text{if}\;x_{\mathrm{int}}^{\min}\leq x\leq x_{\mathrm{int}}^{\max} \end{array} \end{array}$$

9 $$g\left(x_{0},\sigma \right)=\frac{1}{\sigma \sqrt{2\pi }}\exp\left(-\frac{{\left(x-x_{0}\right)}^{2}}{2\sigma^{2}}\right)$$

10 $$\sigma_{1}=0.1\left|x_{\exp}^{\min}-x_{\mathrm{int}}^{\min}\right|\;\text{and}\;\sigma_{2}=0.1\left|x_{\exp}^{\max}-x_{\mathrm{int}}^{\max}\right|$$

where x_exp_^min^ and x_exp_^max^ define the lower and upper boundaries of the experimental window and x_int_^min^ and x_int_^max^ define the borders of the region of interest. This region can be customized by the user; if switched off, the weighting function defaults to a step function, assuming a value of 0 outside the experimental window and 1 inside it.

To validate these features, we have resimulated two previously published, feature-rich ECD spectrum of (*S*)-3-(4-bromophenyl)-3-(trifluoromethyl)­oxirane-2,2-dicarboxylate^33^ and the VCD spectrum of (*S*)-(1,1,1-trifluoro-3-nitropropan-2-yl)­benzene.^34^

The ECD of the epoxide has been calculated in acetonitrile using SMD^35^ implicit solvation theory, using CAM-B3LYP^36^-D4 ^37^ and ωB97X-D4rev as functionals and def2-TZVPP as the common basis set. The resulting spectrum is shown in Figure 3.

**Figure 3:**
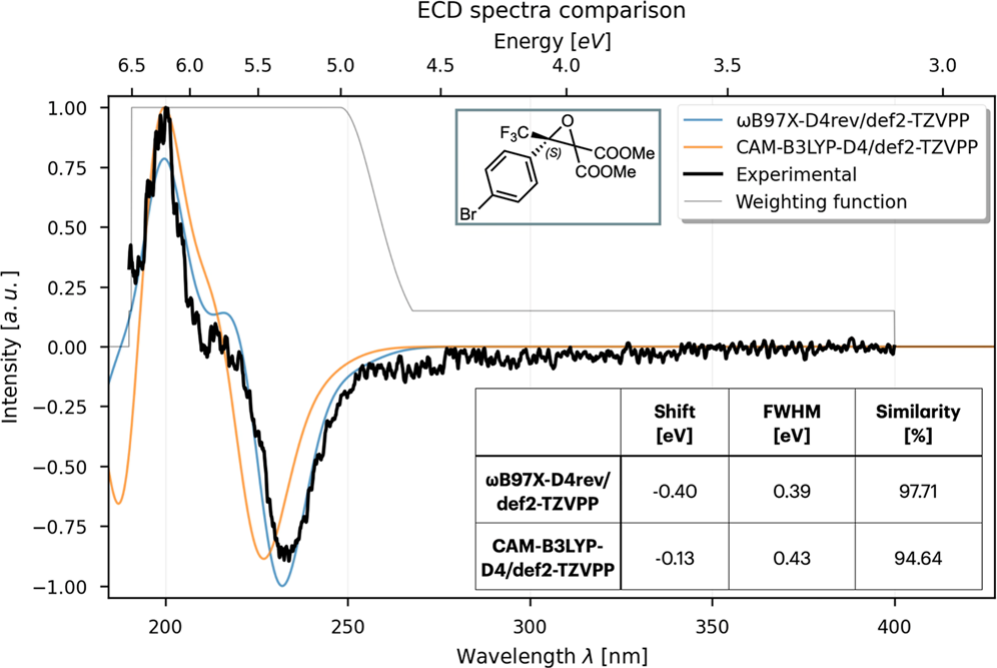
ECD spectrum of (*S*)-3-(4-bromophenyl)-3-(trifluoromethyl)­oxirane-2,2-dicarboxylate. Negative shift values correspond to a blue shift.

The VCD of the nitro-derivative has been simulated in the gas phase using the B3LYP^38^-D4/def2-QZVPPD level, yielding a similarity score of 96% (the result of this study is shown in Supporting Information Figure S5). In all cases, the weighting function is shown to highlight the relevant areas of the spectrum and indicate the ones that have been mostly considered during the optimization process. Further validation on two additional systems with distinct spectral patterns is provided in the Supporting Information.^39^

**Figure 4:**
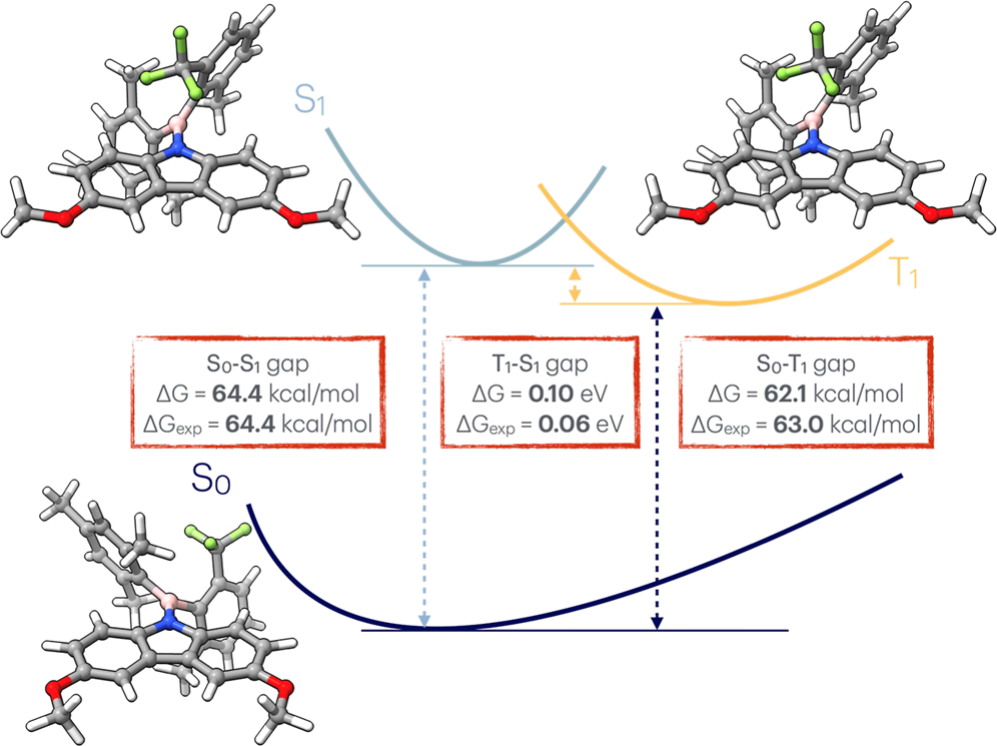
Simplified Jablonski diagram illustrating the singlet and triplet electronic states of a case-study aminoborane.

### 3.4 Estimation of Singlet–Triplet Energy (Δ*E*_S→T_)

Another valuable property of the molecules is the Singlet–Triplet energy gap (Δ*E*_S→T_). This calculation relies on different multiplicities of the whole system. EnAn supports this calculation by allowing users to define both charge and multiplicity for each step in the protocol, enabling workflows for singlet–triplet gaps and other related properties. To demonstrate this capability, we calculated the energy gap for a recently published aminoborane^40^ compound. The protocol was carried out in CPCM­(CHCl_3_) and composed as (i) the optimization of the S_0_ level at the r^2^SCAN-3c level, followed by (ii) a single-point calculation at CAM-B3LYP-D4/def2-TZVPP, (iii) the optimization using ΔSCF^41,42^ with HOMO–LUMO configuration of the α-spin electron for the *S*_1_ electronic state, (iv) followed by a second single-point energy evaluation, and finally, (v) the optimization of the *T*_1_ state, and (vi) its single-point step. A simplified Jablonski diagram summarizing the workflow is shown in Figure 4.

### 3.5 Transition-State Ensemble Refinement

The conformational search is critical not only at a ground-state level but also for the TSs; this analysis can lower the estimated TS energy by up to 10 kcal/mol.^10^ EnAn has been designed to refine TS ensembles, as well. It is possible to insert additional inputs into the protocol definition in each different step. Thus, we created a case study involving two flexible reagents for the classic Diels–Alder reaction. First, a candidate TS geometry was located using Nudged Elastic Band (NEB)^43–45^ at the GFN2-xTB level using ORCA, then optimized toward the saddle point at the r^2^SCAN-3c level, and finally, its electronic energy was evaluated at the ωB97X-3c^28^ level. From this geometry, an ensemble of possible TSs has been generated using GOAT, and this massive group of conformers (673) has been processed into EnAn with the following protocol: (i) creation of 30 clusters at the GFN2-xTB energy level, (ii) constrained optimization at r^2^SCAN-3c freezing the four reacting atoms, (iii) the actual optimization toward the saddle point at the same level, and (iv) the final electronic energy calculation at ωB97X-3c, in order to obtain comparable energy values.

This refinement decreased the TS energy by 4.8 kcal/mol (Figure 5). This example demonstrates that even small changes in some peripheral substituents of the reactants or catalyst might significantly alter reaction pathway energies, potentially affecting the selectivity of the process studied.

**Figure 5:**
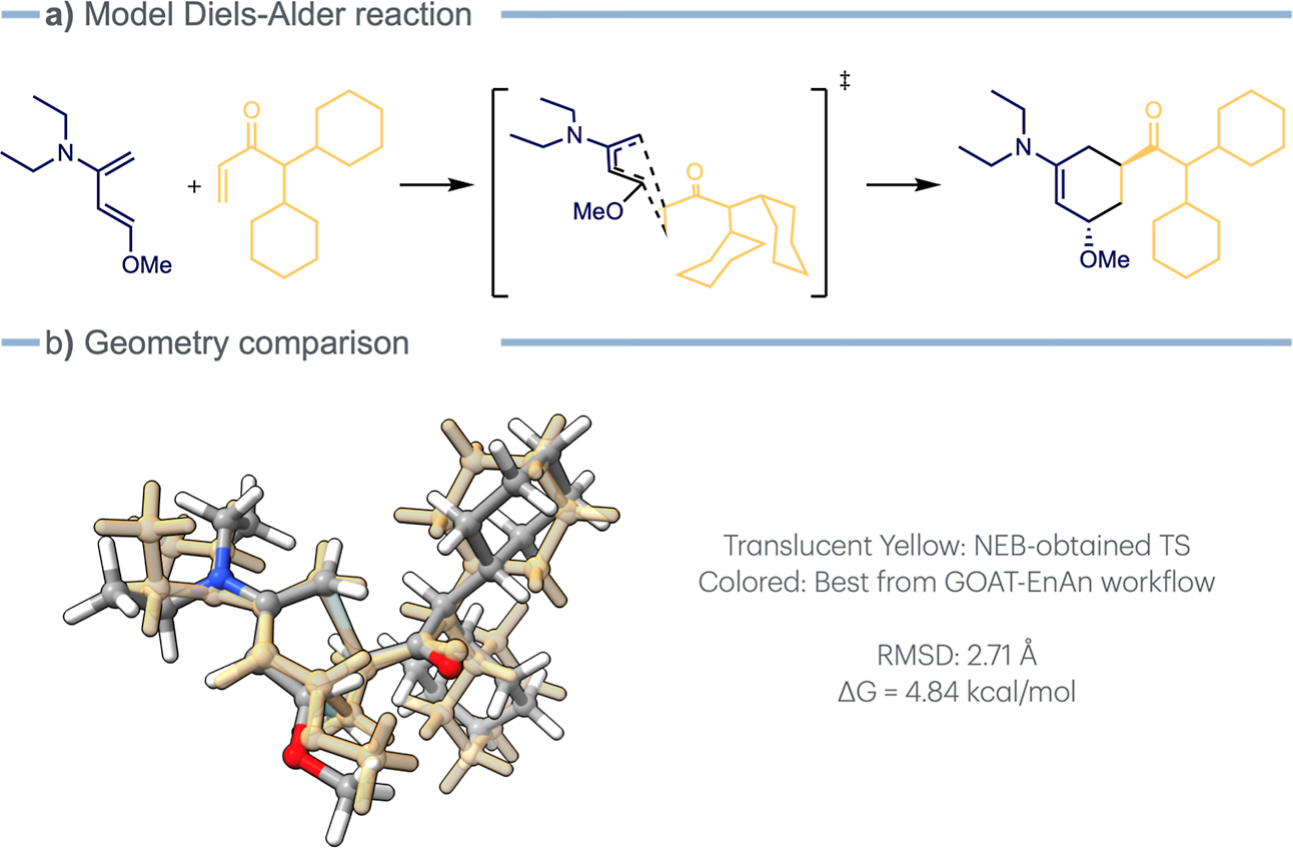
(a) Case study Diels–Alder reaction. (b) Comparison between the TS obtained through the NEB workflow (translucent yellow) and the one refined through the GOAT-EnAn workflow.

## 4 Conclusions

In this work, we present a computational framework designed to automate the management of conformational ensembles by interfacing with multiple QM engines. The sheer size of such ensembles often renders manual processing intractable, necessitating automated solutions.

A distinct advantage of this software is that it enables researchers, regardless of their programming expertise, to define reproducible text-based protocols for selecting representative conformers. This filtering process is driven by pairwise comparisons against user-defined thresholds for rotational constants and electronic energies alongside PCA-guided clustering.

The versatility of the tool was demonstrated through diverse case studies: (i) the optimization and refinement of ensembles for medium to large, highly flexible scaffolds; (ii) ensemble reduction via PCA clustering; (iii) the simulation of VCD and ECD spectra for chiral systems; (iv) the assessment of singlet–triplet energy gaps in a recently reported system; and (v) the refinement of TS ensembles for model reactions bearing flexible substituents. In all scenarios, the pipeline successfully streamlined the workflow, yielding robust results with minimal user intervention.

## Supplementary Material



## Data Availability

The source code is available on GitHub at https://github.com/andre-cloud/ensemble_analyzer and archived at DOI: 10.5281/zenodo.18255912.

## Notes

Installation instructions and system requirements; theoretical background on conformational ensemble refinement and spectral property calculations; comprehensive usage guide with step-by-step tutorials; and detailed computational protocols and parameters for all case studies (PDF)
