# Supplementary material for: Ensemble Analyzer: An Open-Source Python Framework for Automated Conformer Ensemble Refinement
Source: J Chem Inf Model. 2026 Apr 21;66(9):5018–25. doi: 10.1021/acs.jcim.6c00273 (PMC13169348; doi:10.1021/acs.jcim.6c00273)
Supplement: Supplementary file 1 [file ci6c00273_si_001.pdf]

Supporting Information
Ensemble Analyzer: An Open-Source Python Framework for Automated Conformer Ensemble Refinement

version 1.0.0

Andrea Pellegrini,* Paolo Righi,
Andrea Mazzanti, Michele Mancinelli

Department of Industrial Chemistry "Toso Montanari", Alma Mater Studiorum University of Bologna, Via P. Gobetti 85, 40129 Bologna, Italy

April 9, 2026

TABLE OF CONTENTS

1 Theoretical Background **S7**
- 1.1 Conformational Pruning Strategy . . . S8
- 1.2 Invariant Clustering Algorithm . . . S8
- 1.3 Thermochemistry: The qRRHO Approximation . . . S9
- 1.4 Boltzmann Weighting and Spectra . . . S10

2 Installation and Setup **S13**
- 2.1 Prerequisites . . . S13
- 2.2 Python Environment Setup . . . S13
- 2.3 External QM software . . . S14
- 2.4 Verification . . . S14

3 Analysis Workflow **S17**
- 3.1 Input & Initialization . . . S17
- 3.2 The Refinement Loop . . . S17
- 3.3 Finalization and Output . . . S19

4 Auxiliary CLI Tools **S21**
- 4.1 Installation Check (`enan_check`) . . . S21
- 4.2 Protocol Wizard (`enan_protocol_wizard`) . . . S21
- 4.3 Spectra Regrapher (`enan_regraph`) . . . S22
- 4.4 Average Energy Analyzer (`enan_get_energies`) . . . S22
- 4.5 Graph Editor (`enan_graph_editor`) . . . S23

5 Protocol Configuration Guide **S25**
- 5.1 Core Computational Keywords . . . S25
- 5.2 Advanced Calculation Control . . . S25
- 5.3 Refinement and Pruning Settings . . . S26

6 CLI Tools Reference **S27**

7 Quickstart **S29**

8 Example 1: Multi-Level Ensemble Refinement **S31**
- 8.1 Clustering Impact Analysis . . . S32

9 Example 2: VCD and ECD Spectra Simulation **S37**

10 Example 3: Singlet-Triplet Energy Gap (ΔE S→T) **S43**

11 Example 4: Transition State Ensemble Refinement **S47**

LIST OF FIGURES

S1 Scheme of the workflow of EnAn S18

S2 Flexible molecules used as case study for the clustering analysis. S33
S3 Variation of ΔE_{av} loss as a function of the conformer's retention. S34
S4 Trade-off function between accuracy (in red) and wall time (in blue) as a function of the retention rate. Performed on 44 Intel® Xeon® CPU cores. S35

S5 Convoluted VCD obtained with optimized parameters. S38
S6 Convoluted ECD obtained with optimized parameters. S39
S7 Automated fitting of the simulated UV-Vis spectrum against the experimental reference. S40
S8 Comparison of the automated ECD spectral fitting (a) without and (b) with the application of the weighting function. S41

S9 Simplified Jablonski diagram S45

S10 Summary of the TS conformational search. S48

CHAPTER

ONE

THEORETICAL BACKGROUND

Ensemble Analyzer (EnAn) streamlines the processing of conformational ensembles by integrating geometric filtering, unsupervised clustering, and advanced thermochemical corrections.

To contextualize the capabilities of EnAn, we compared its architecture with other state-of-the-art computational tools, namely Autobench, CENSO, and autodE (Table S1). While these established workflows excel at specific tasks—such as systematic benchmarking, hierarchical thermochemical refinement, and automated transition state location—they generally rely on rigid, predefined methodologies. We consider the underlying quantum mechanical engines (e.g., Gaussian and ORCA) strictly as computational calculators; consequently, EnAn is designed not as a fixed methodology, but as a highly modular, automated interface. Its primary objective is to overcome the inherent rigidity of current pipelines by providing code-free automation through the `enan_protocol_wizard` command. Unlike the aforementioned tools, EnAn enables the dynamic variation of charge and multiplicity within a single protocol, the flexible positioning of PCA-based clustering at any workflow stage, and the straightforward injection of additional input parameters into the QM engines. Furthermore, it streamlines post-processing by integrating automated spectral fitting (via RMSD minimization for shift and FWHM) and generating publication-ready figures through an interactive text user interface (TUI).

Feature	Autobench	CENSO	autodE	Ensemble Analyzer
**Architectural Focus**	Fixed methodology (Benchmarking)	Fixed methodology (Refinement)	Fixed methodology (TS Search)	Modular automated interface
**Protocol Composition**	Rigid workflow	Predefined hierarchy	Fixed (Reactants/Products/TS)	Modular (user-definable computational jobs)
**Charge & Multiplicity**	Fixed per single execution	Fixed for the entire ensemble	Molecule/state specific	Dynamic intra-protocol variation
**Ensemble Management**	RMSD and frequency-based pruning	Energy sorting and pruning	Internal conformational exploration	Dynamic positionable PCA clustering and pruning
**QM engine Parameter Injection**	Global via config file	Global via predefined input files	Predefined keywords/templates	Direct and flexible injection on a per-step basis
**Spectral Analysis**	Not applicable	Calculation only	Not applicable	Automated fitting (RMSD minimization on shift/FWHM)
**Graphical Output**	Not applicable / Basic	Basic spectral plots	2D energy profiles	Publication-ready figures via interactive TUI

Table S1: Comparison of software architectures and operational capabilities for computational chemistry workflows.

Job Type	thrG_{max} (kcal/mol)	thr$G$ (kcal/mol)	thr$B$ (cm^{-1})
SP	6.0	0.25	1×10^{-5}
OPT	4.0	0.5	5×10^{-5}
FREQ	3.75	0.5	5×10^{-5}
OPT+FREQ	3.5	0.5	5×10^{-5}

Table S2: Default Job-Dependent Energy and Geometric Thresholds. thrG_{max}: Maximum energy window for retention; thr$G$: Energy tolerance for duplicate detection; thr$B$: Rotational constant tolerance for geometric equivalence. All parameters are user-configurable.

1.1 Conformational Pruning Strategy

A critical bottleneck in conformational analysis is the redundancy of structures generated by stochastic search algorithms. EnAn implements a computationally efficient **dual-filter** to remove duplicates without requiring expensive RMSD (Root-Mean-Square Deviation) superposition for every pair.

1.1.1 Energy Filtering (The "Window")

The first tier of filtering acts on the electronic energy (ΔE). Given the global minimum found so far (E_{min}), any conformer i is discarded if:

$$(E_i - E_{min}) > \text{thr}G_{\text{max}}$$

This ensures that only chemically relevant conformers (accessible at thermal equilibrium) are processed.

1.1.2 Geometric Filtering: Rotational Constants

To identify duplicates, EnAn utilizes **Rotational Constants** as geometric descriptors. Rotational constants depend on the principal moments of inertia (I) and are invariant to translation and rotation of the molecular frame.

Two conformers i (check) and j (reference) are defined as **identical** if they satisfy *both* conditions:

1. **Energy Equivalence**: $|E_i - E_j| <$ thrG
2. **Geometric Equivalence**: $|B_i - B_j| <$ thrB

Where B is the scalar norm of the rotational constant vector. This method reduces the complexity from $O(N^2)$ RMSD alignments to simple scalar comparisons.

Energy thresholds are automatically adjusted based on the calculation type to balance retention and computational cost. Single-point calculations employ wider energy windows (thr$G_{\text{max}} = 6.0$ kcal/mol) to retain conformers that may stabilize upon optimization, while optimization and frequency calculations use progressively tighter cutoffs (thr$G_{\text{max}} = 3.5$-4.0 kcal/mol). Similarly, the energy tolerance for duplicate detection (thr$G$) and the rotational constant tolerance (thr$B$) vary by job type. Default values are summarized in Table S2; all parameters are user-configurable through the protocol definition file.

> **Note**: An invariant RMSD based on EDM eigenvalues is calculated for logged duplicates.

1.2 Invariant Clustering Algorithm

For structural classification and ensemble reduction, EnAn moves away from Cartesian coordinate dependence to avoid alignment issues. It employs the **Spectrum of the Euclidean Distance Matrix (EDM)**.

1.2.1 The Euclidean Distance Matrix

For a molecule with N atoms, the EDM (**D**) is an $N \times N$ symmetric matrix where each element represents the squared Euclidean distance between atom i and atom j:

$$D_{ij} = ||\mathbf{r}_i - \mathbf{r}_j||^2$$

1.2.2 Eigenvalues as Descriptors

The set of eigenvalues $\{\lambda_1, \lambda_2, \ldots, \lambda_N\}$ of the matrix **D** is invariant to:

- Translation
- Rotation
- Atom numbering (permutations, if sorted)

This eigenvalue vector serves as the input feature vector for **Principal Component Analysis (PCA)**. The resulting Principal Components (PCs) are then fed into a **K-Means** clustering algorithm. The optimal number of clusters (k) is automatically determined using the **Silhouette Score**.

1.3 Thermochemistry: The qRRHO Approximation

Standard Rigid-Rotor Harmonic Oscillator (RRHO) approximations often fail for flexible molecules with low-frequency vibrational modes (< 100 cm^{-1}), leading to infinite entropy errors.

EnAn implements the **Quasi-Rigid Rotor Harmonic Oscillator (qRRHO)** approximation described by Grimme (2012). This method interpolates between rigid-rotor and harmonic oscillator limits using a damping function $w(\nu)$:

$$w(\nu) = \frac{1}{1 + (\omega_0/\nu)^\alpha}$$

where ω_0 is the cut-off frequency (default: 100 cm^{-1}) and α is the damping power (default: 4).

Thermodynamic properties are calculated assuming a non-interacting particle ensemble (Ideal Gas approximation). The total Internal Energy (U) and Entropy (S) are obtained by summing the contributions from translational, rotational, electronic, and vibrational degrees of freedom.

1.3.1 Internal Energy (U)

The thermal contribution to the internal energy is calculated as:

$$U_{\text{tot}} = U_{\text{trans}} + U_{\text{rot}} + U_{\text{vib}}$$

- **Translational Energy** (U_{trans}): Derived from the equipartition theorem for 3 degrees of freedom: $U_{\text{trans}} = \frac{3}{2}k_BT$
- **Rotational Energy** (U_{rot}): Depends on the molecular geometry (linearity):
  - **Non-linear**: $\frac{3}{2}k_BT$ (3 rotational degrees of freedom).
  - **Linear**: k_BT (2 rotational degrees of freedom).
- **Vibrational Energy** (U_{vib}): EnAn employs the **qRRHO** (Quasi-Rigid Rotor Harmonic Oscillator) scheme to correct the unphysical behavior of low-frequency modes. The energy is interpolated between a Rigid-Rotor-like term (for low frequencies) and the Harmonic Oscillator term (for high frequencies):

$$U_{\text{vib}} = \sum_{i}^{N_{\text{modes}}} \left(w(\nu_i) U_{\text{HO}}(\nu_i) + [1 - w(\nu_i)] \frac{1}{2} k_B T \right)$$

  Where:

- $U_{\text{HO}}(\nu) = \frac{h\nu c}{e^{\frac{h\nu c}{k_B T}}-1}$ is the standard harmonic oscillator thermal energy.
- $w(\nu)$ is the damping function.

1.3.2 Entropy (S)

Total entropy is defined as $S_{\text{tot}} = S_{\text{trans}} + S_{\text{rot}} + S_{\text{el}} + S_{\text{vib}}$.

- **Translational Entropy (S_{trans})**: Calculated using the **Sackur-Tetrode equation**: $S_{\text{trans}} = k_B \left(\ln \left(\left(\frac{2\pi MW k_B T}{N_A h^2} \right)^{3/2} \frac{k_B T}{P} \right) + \frac{5}{2} \right)$
- **Rotational Entropy (S_{rot})**: Calculated within the **Rigid Rotor (RR)** approximation:
  - **Non-linear**: $S_{\text{rot}} = k_B \left(\ln \left(\frac{\sqrt{\pi} T^{3/2}}{\sigma \sqrt{\theta_A \theta_B \theta_C}} \right) + \frac{3}{2} \right)$
  - **Linear**: $S_{\text{rot}} = k_B \left(\ln \left(\frac{T}{\sigma\theta} \right) + 1 \right)$

  Where $\theta_x = \frac{hcB_x}{k_B}$ are the characteristic rotational temperatures derived from the rotational constants B, and σ is the symmetry number, considered for all molecules as 1 (thus with a C_1 point group).
- **Electronic Entropy (S_{el})**: Determined solely by the spin multiplicity (m): $S_{\text{el}} = k_B \ln(m)$
- **Vibrational Entropy (S_{vib})**: Following the method by Grimme. entropy is interpolated between the Harmonic Oscillator limit (S_{HO}) and a free-rotor entropy term ($S_{\text{rot}}^{\text{Grimme}}$) using the damping function $w(\nu)$:

$$S_{\text{vib}} = \sum_{i}^{N_{\text{modes}}} \left(w(\nu_i) S_{\text{HO}}(\nu_i) + [1 - w(\nu_i)] S_{\text{rot}}^{\text{Grimme}}(\nu_i) \right)$$

  The rotor contribution S_{rot}^{Grimme} considers the effective moment of inertia of the vibrational mode (μ) and the average molecular rotational constant (B_{av}):

$$S_{\text{rot}}^{\text{Grimme}} = \frac{1}{2} k_B \left(1 + \ln \left(\frac{8\pi^3 \mu B_{\text{av}}}{\mu + B_{\text{av}}} \frac{k_B T}{h^2} \right) \right)$$

1.3.3 Enthalpy (H) and Gibbs Free Energy (G)

The final thermodynamic potentials are assembled as follows:

$$H = E_{\text{SCF}} + \text{ZPVE} + U_{\text{tot}} + k_B T$$

$$G = H - T S_{\text{tot}}$$

Where:

- E_{SCF} is the electronic energy from the QM calculation.
- ZPVE (Zero Point Vibrational Energy) is the sum of $\frac{1}{2} h\nu c$ for all real frequencies.
- $k_B T$ is the PV work term for an ideal gas ($H = U + PV$).

1.4 Boltzmann Weighting and Spectra

Final spectral properties are computed as a Boltzmann-weighted average of the individual active conformers.

1.4.1 Population Calculation

The probability p_i of finding the system in conformer i at temperature T is derived from the relative Gibbs Free Energy (ΔG_i):

$$p_i = \frac{e^{-\Delta G_i/k_B T}}{\sum_j e^{-\Delta G_j/k_B T}}$$

1.4.2 Spectral Convolution

EnAn distinguishes between vibronic and electronic spectra for the convolution function $f(\nu, \nu_0, \sigma)$:

1. **Vibronic Spectra (IR, VCD)**: Use a **Lorentzian** line-shape function to model lifetime broadening:

$$f_{\text{Lorentz}}(x; x_0, \gamma) = \frac{\text{FWHM}^2}{\text{FWHM}^2 + 4(x - x_0)^2}$$

2. **Electronic Spectra (UV-Vis, ECD)**: Use a **Gaussian** line-shape function to model inhomogeneous broadening:

$$f_{\text{Gauss}}(x; x_0, \sigma) = \frac{1}{\sigma\sqrt{2\pi}} e^{-\frac{1}{2}\left(\frac{x-x_0}{\sigma}\right)^2}$$

The final intensity $I_{\text{total}}(\nu)$ is the weighted sum:

$$I_{\text{total}}(\nu) = \sum_{i}^{N_{\text{conf}}} p_i \sum_{k}^{N_{\text{modes}}} I_{i,k} \cdot f(\nu, \nu_{i,k}, \text{FWHM})$$

CHAPTER

TWO

INSTALLATION AND SETUP

Ensemble Analyzer (EnAn) is a Python-based framework that acts as a high-level driver for quantum mechanical calculations. Therefore, a complete installation involves setting up the Python environment and ensuring the external QM software is correctly linked.

2.1 Prerequisites

Before installing the package, ensure your system meets the following requirements:

- **Operating System**: Linux (recommended), macOS, or Windows.
- **Python**: Version 3.11 or higher.
- **QM Engine**: A valid installation of ORCA (recommended) and/or Gaussian.

2.2 Python Environment Setup

We strongly recommend using **Conda** (via Anaconda or Miniconda) to manage dependencies and avoid conflicts with system libraries.

2.2.1 Create a Virtual Environment

```
# Create a new environment named 'enan'
conda create -n enan python>=3.11

# Activate the environment
conda activate enan
```

2.2.2 Install the package

You can install EnAn directly using `pip`. This automatically resolve core dependencies.

```
pip install ensemble-analyzer
```

For developers who want to modify/add some feature

```
git clone
cd ensemble_analyzer
pip install -e .
```

2.3 External QM software

EnAn does not include quantum chemistry codes; it drives them. You must configure at least one calculator.

2.3.1 Option A: ORCA (Recommended)

1. **Download & install**: obtain the ORCA binaries directly from the official ORCA forum.
2. **System Path**: ensure the orca executable is in your system `$PATH` variable.

```
which orca
# Should return a path, e.g., /opt/orca/orca
```

3. **Environment Variable (Crucial)**: EnAn requires the specific ORCA version to handle output parsing correctly. You *must* export the `ORCAVERSION` variable. Add the following lines to your shell configuration file (e.g. `~/.bashrc` or `~/.zshrc`):

```
# EnAn specific variable
export ORCAVERSION="6.1.0"
```

Failure to set `ORCAVERSION` will cause the calculator and the parser to crash during the protocol execution.

2.3.2 Option B: Gaussian

If you have a licensed version of Gaussian installed:

1. Ensure the standard Gaussian environment variables are loaded (`g09.profile` or `g16.profile`)
2. EnAn will detect the `g16`or `g09`executable automatically from the path.

2.4 Verification

To verify that EnAn is correctly installed and linked to your QM software (ORCA/Gaussian), simply run the built-in diagnostic tool:

```
enan_check
```

This command will check Python dependencies, system paths, and the ORCAVERSION environment variable. The expected output looks as follows:

```
$ enan_check
Running Ensemble Analyzer Installation Check...


-------------------- 1. Checking Python Dependencies --------------------
[PASS] Library found: numpy
[PASS] Library found: scipy
[PASS] Library found: matplotlib
[PASS] Library found: ase
[PASS] Library found: numba
[PASS] Library found: sklearn
[PASS] Ensemble Analyzer package is installed and importable.

-------------------- 2. Checking ORCA Configuration --------------------
[PASS] ORCA executable found at: /opt/orca/6.1.0/orca
```

(continues on next page)

(continued from previous page)

```
[PASS] ORCAVERSION environment variable is set to: 6.1.0

-------------------- 3. Checking Gaussian Configuration --------------------
[PASS] Gaussian 16 found at: /opt/gaussian/16A03/g16

--------------------------------------------------
SUCCESS: Installation looks correct! You are ready to run EnAn.
```

CHAPTER

THREE

ANALYSIS WORKFLOW

This guide details the **internal workflow** of **Ensemble Analyzer**, illustrating how data flow from the initial input through the refinement pipeline to the final property generation.

3.1 Input & Initialization

The workflow begins by parsing the command-line arguments and initializing the core managers.

3.1.1 Data Loading

The `launch.py` entry point triggers the loading phase:

- **Ensemble Loading**: The geometry file (e.g., `.xyz`) is parsed into a list of `Conformer` objects.
- **Protocol Loading**: The JSON protocol is deserialized into a list of `Protocol` objects, defining the sequence of computational steps (e.g., Optimization → Frequency → Single Point).
- **Configuration**: Global settings (temperature, CPU count, solvent models) are loaded into the `CalculationConfig` object.

3.1.2 Initial Analysis

Before starting the refinement loop, if the ensemble contains sufficient structures ($N > 30$), an initial **Principal Component Analysis (PCA)** is performed on the input geometries to visualize the starting conformational space coverage.

3.2 The Refinement Loop

The core logic is handled by the `CalculationOrchestrator`, which iterates through each step defined in the `protocol.json`. For each protocol step, the `ProtocolExecutor` performs the operations schematically shown in Figure S1:

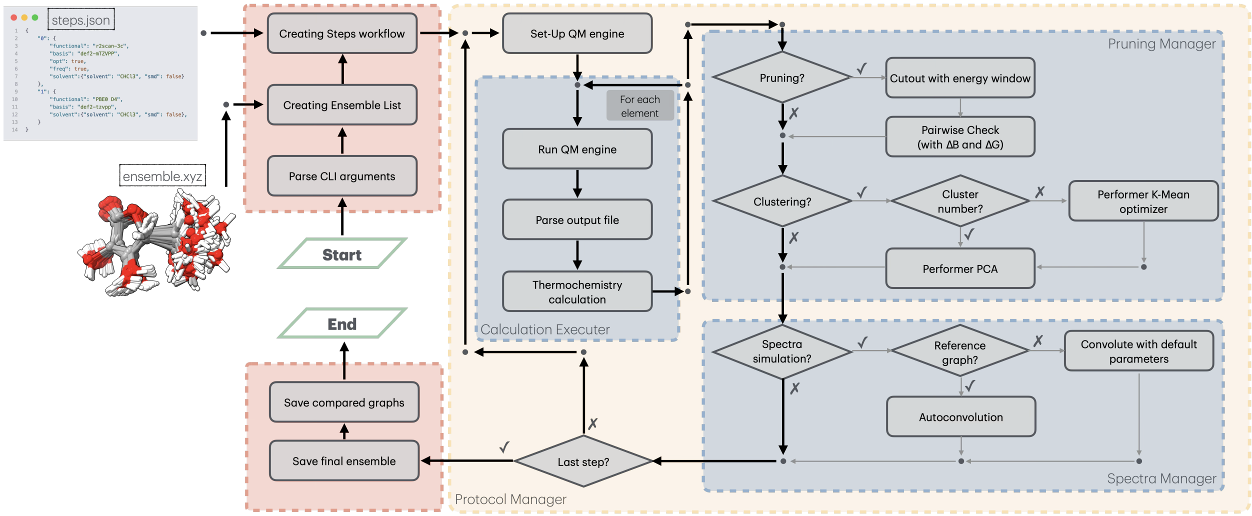

Figure S1: Scheme of the workflow of EnAn

3.2.1 Quantum Mechanical Calculations

For every active conformer that has not yet been calculated at the current level:

1. **Input Generation**: The `CalculationExecutor` generates input files for the external QM engine (ORCA or Gaussian).
2. **Execution**: The calculation is run (Optimization, Frequency, or Single Point).
3. **Parsing**: Results (Energy, Geometry, Dipoles, Rotational Constants, Vibrational Frequencies) are parsed and stored in the `EnergyStore` of the conformer.
4. **Checkpointing**: The `CheckpointManager` saves the state atomically after every calculation to prevent data loss.

3.2.2 Pruning Stage

After the calculation phase, the `PruningManager` filters the ensemble to remove high-energy structures and geometrically redundant conformers. This step is critical to reduce computational cost for subsequent, more expensive steps.

The pruning logic consists of two main filters:

1. **Energy Window Filtering**: Conformers with a relative energy above the threshold ($\Delta E > \text{thr}G_{\text{max}}$) are immediately deactivated.
   - *Parameter*: `thrGMAX` (defined in `threshold.json` or protocol).
2. **Geometric Filtering (Duplicate Removal)**: Instead of computationally expensive RMSD alignments, EnAn uses **Rotational Constants** (B) and **Electronic Energy** (E) as descriptors to identify duplicates. Two conformers i and j are considered identical if: $|\Delta E_{ij}| < \text{thrG} \quad \wedge \quad |\Delta B_{ij}| < \text{thrB}$
   - *Parameters*: `thrG` (Energy tolerance), `thrB` (Rotational constant tolerance).
   - *Validation*: For logged duplicates, an RMSD based on the **Euclidean Distance Matrix (EDM)** eigenvalues is calculated for verification.

Note: Pruning can be disabled for specific steps by setting `"no_prune": true` in the protocol.

3.2.3 Clustering & Analysis

If clustering is enabled in the protocol (`"cluster": true` or specific integer), the `ClusteringManager` performs an unsupervised structural analysis to group conformers and optionally reduce the ensemble.

The workflow utilizes **invariant features** to avoid coordinate alignment issues:

1. **Feature Extraction**: The eigenvalues of the Euclidean Distance Matrix (EDM) are computed for each conformer. These are invariant to translation and rotation.
2. **PCA (Principal Component Analysis)**: Dimensionality reduction is applied to the EDM eigenvalues.
3. **K-Means Clustering**:
   - If a specific number of clusters is provided, K-Means is run directly.
   - If set to `auto`, the optimal number of clusters (k) is determined via **Silhouette Score** analysis (scanning $k = 2$ to $k = N_{\text{conf}} \times 0.8$).
4. **Ensemble Reduction**: The ensemble is reduced by retaining only the representative conformer (lowest energy) from each cluster.

3.2.4 Spectral Generation

The final stage of a protocol step involves generating continuous spectra from discrete transitions. This is handled by the `graph` module.

- **Vibronic Spectra (IR, VCD)**: Convolved using **Lorentzian** functions.
- **Electronic Spectra (UV-Vis, ECD)**: Convolved using **Gaussian** functions.

Population Weighting: All spectra are weighted based on the Boltzmann population of the conformers, calculated using the relative Gibbs Free Energy (ΔG) at the specified temperature (T).

3.3 Finalization and Output

Once all protocol steps are completed, the `CalculationOrchestrator` finalizes the workflow:

3.3.1 Data Export

- `final_ensemble.xyz`: A multi-structure XYZ file containing all surviving conformers, sorted by energy.
- `checkpoint.json`: A complete state file allowing for restarts or post-processing analysis.

3.3.2 Comparative Plotting

The `plot_comparative_graphs` module automatically generates overlay plots (e.g., `IR_comparison.png`, `UV_comparison.png`). These plots visualize the evolution of the computed spectra across the different protocol levels (e.g., comparing the spectrum after `SP` vs `OPT+FREQ`), allowing for quick assessment of convergence and method dependence.

3.3.3 Reporting

A summary table is printed to the log (`output.out`), detailing:

- Final energies (E, H, G) and ZPVE.
- Boltzmann populations.
- Total elapsed time and final retention rate.

CHAPTER

FOUR

AUXILIARY CLI TOOLS

In addition to the main `ensemble_analyzer` driver, the package provides a suite of command-line interface (CLI) tools to assist with setup, pre-processing configuration, and post-processing analysis.

4.1 Installation Check (`enan_check`)

A diagnostic tool to verify the installation of python dependencies and the linkage to external QM software (ORCA/Gaussian).

Usage:

```
enan_check
```

Checks performed:

- Presence of core Python libraries (`numpy`, `ase`, `scipy`, etc.).
- Availability of the orca executable in the system PATH.
- Correct configuration of the `ORCAVERSION` environment variable.
- Availability of Gaussian executables (`g16` or `g09`).

4.2 Protocol Wizard (`enan_protocol_wizard`)

An interactive Text User Interface (TUI) to generate the `protocol.json` file. It guides the user through the creation of computational steps without needing to manually edit JSON files.

Usage

```
enan_protocol_wizard
```

Features:

- Three Configuration Levels:
  - Basic: Essential parameters only.
  - Intermediate: Adds constraints and internal coordinate monitoring.
  - Advanced: Full control over energetic thresholds (`thrG`, `thrB`, *etc.*) and pruning settings.
- Fuzzy Search: Quickly find functionals and basis sets from the internal database (for ORCA).

The basic and Intermediate levels allow to less-expert users to create from scratch a protocol file, using the default values of some configuration.

4.3 Spectra Regrapher (`enan_regraph`)

Regenerates spectral plots (IR, VCD, UV, ECD) without re-running the quantum calculations. This is useful when you want to adjust convolution parameters (FWHM, Shift) or change the plotting units (nm vs eV) after the main run.

Usage:

```
enan_regraph [protocol_ids ...] [options]
```

Arguments:

- `protocol_ids`: (Required) List of protocol numbers to regraph (e.g., 0 1 2).

Options:

- `-rb`, `--read-boltz <int>`: Use Boltzmann populations from a specific protocol step instead of recalculating them.
- `-no-nm`: Do not save graphs with nanometer (nm) x-axis (saves only default units).
- `-w`, `--weight`: Overlay the weighting function used for comparison on the plot.
- `--disable-color`: Disable colored terminal output.

Example: Recalculate spectra for step 1 using new settings in settings.json:

```
enan_regraph 1
```

4.4 Average Energy Analyzer (`enan_get_energies`)

Calculates weighted average energies (E, H, G) for the ensemble at a specific temperature. It allows for thermodynamic recalculations (different T or P) and arithmetic operations between protocol steps.

Usage:

```
enan_get_energies [options]
```

Core Options:

- `-T`, `--temp <float>`: Temperature in Kelvin for recalculation (default: 298.15 K).
- `-o`, `--output <file>`: Output log filename.
- `--pressure <float>`: Pressure in kPa (default: 101.325).

Thermo Parameters:

- `--cut-off <float>`: qRRHO cut-off frequency in cm^{-1} (default: 100.0).
- `--alpha <int>`: qRRHO damping factor (default: 4).
- `--linear`: Treat molecules as linear rigid rotors.

Math Operations:

- `--sub <p1> <p2>`: Calculate difference Avg(Protocol 1) - Avg(Protocol 2).
- `--add <p1> <p2>`: Calculate sum Avg(Protocol 1) + Avg(Protocol 2).

Example: Compare energies between protocol 2 and 1 at 310K:

```
enan_get_energies -T 310 --sub 2 1
```

4.5 Graph Editor (`enan_graph_editor`)

A tool to modify the appearance of the generated Matplotlib plots (saved as .pickle files). It supports both an interactive TUI and a batch mode for automated editing.

Usage:

```
enan_graph_editor <pickle_file> [options]
```

Interactive Mode (TUI)

Simply run without arguments to enter the interactive menu:

```
enan_graph_editor IR_comparison.pickle
```

Allows renaming labels, changing colors, line styles, widths, and transparency.

Batch Mode

Use the –batch flag to apply changes via command line arguments.

Options:

- `--list`, `-l`: List current legend labels and exit.
- `--rename`, `-r <old> <new>`: Rename a legend label.
- `--color`, `-c <label> <color>` : Change line color.
- `--linestyle`, `-ls <label> <style>`: Change line style (e.g., -, –, :).
- `--format`, `-f`: Output format (png, pdf, svg, pickle).

Example: Rename a protocol and change its color to red, saving as PNG:

```
enan_graph_editor IR_comparison.pickle --batch \
            --rename "Protocol 1" "B97-3c" \
            --color "B97-3c" "red" \
            --format png
```

CHAPTER

FIVE

PROTOCOL CONFIGURATION GUIDE

The protocol file is the core configuration for **Ensemble Analyzer**. It is structured as a JSON dictionary where each key (e.g., `"0"`, `"1"`) represents a sequential computational step.

5.1 Core Computational Keywords

These parameters define the level of theory and the type of calculation to be performed by the QM engine.

- **`functional`** (str, *Required*) The DFT functional or semi-empirical method to use (e.g., `"B97-3c"`, `"wB97X-D4"`, `"xtb"`).
- **`basis`** (str) The basis set definition. If using composite methods (like r^2`SCAN-3c`), this is automatically handled or can be omitted.
- **`opt`** (bool) If `true`, performs a geometry optimization for the current step.
- **`freq`** (bool) If `true`, performs a frequency calculation. This enables vibrational analysis and qRRHO thermochemical corrections.
- **`charge`** (int) The total charge of the system (default: `0`).
- **`mult`** (int) The spin multiplicity of the system (default: `1`).
- **`solvent`** (dict) Configuration for implicit solvation models.
  - `solvent` (str): Name of the solvent (e.g., `"water"`, `"chcl3"`).
  - `smd` (bool): If `true`, uses the **SMD** solvation model; otherwise, uses **CPCM**.

5.2 Advanced Calculation Control

- **`add_input`** (str) Additional keywords or blocks passed directly to the external QM engine (ORCA/Gaussian) input file. **No sanity check performed**.
- **`read_orbitals`** (int) Specifies the index of a previous step to read orbitals/guess from (e.g., `"0"`). Useful for SCF convergence in difficult cases.
- **`skip_opt_fail`** (bool) If `true`, conformers that fail to converge during optimization are automatically deactivated instead of crashing the workflow.
- **`monitor_internals`** (list) A list of atom indices to track specific internal coordinates in the log output.
  - *Example:* `[[0, 1], [2, 3, 4]]` monitors a bond length and an angle.
- **`block_on_retention_rate`** (bool) If `true`, the program will halt execution if the number of surviving conformers drops below a safety threshold (default 20%), preventing total loss of the ensemble.

5.3 Refinement and Pruning Settings

- **`cluster`** (int | bool) Controls the unsupervised clustering of conformers.
  - If an **integer > 1**: Performs K-Means clustering to reduce the ensemble to that exact number of structures.
  - If **`true`**: Performs clustering with automatic detection of the optimal number of clusters (k).
- **`no_prune`** (bool) If `true`, completely disables energy and geometric pruning for this specific step.
- **`thrG`** / **`thrB`** (float) Overrides the default thresholds for identifying duplicates:
  - `thrG`: Maximum energy difference (ΔE) [kcal/mol].
  - `thrB`: Maximum difference in Rotational Constants (ΔB) [cm^{-1}].
- **`thrGMAX`** (float) Overrides the maximum energy window cut-off. Conformers with $\Delta E >$ thrGMAX (relative to the global minimum) are discarded.

CHAPTER

SIX

CLI TOOLS REFERENCE

The main driver `ensemble_analyzer` accepts various command-line arguments to configure the runtime environment, physical constants, and post-processing options.

Input & Flow Control

- **`-e`**, **`--ensemble`** (path) The input XYZ file containing the initial conformational ensemble.
- **`-p`**, **`--protocol`** (path) Path to the `protocol.json` file. If omitted, the internal default protocol is used.
- **`--restart`** Resumes the calculation from the last completed protocol step using the generated `checkpoint.json`.
- **`-o`**, **`--output`** (str) Name of the main log file (default: `output.out`).

Physics & Thermodynamics

- **`-T`**, **`--temperature`** (float) Temperature in Kelvin used for Boltzmann weighting and thermochemical analysis (default: `298.15` K).
- **`-P`**, **`--pressure`** (float) Pressure in kPa (default: `101.325`).
- **`--linear`** Flag to indicate that the molecules should be treated as linear rigid rotors (affects entropy calculations).
- **`-cut-off`**, **`--cut-off`** (float) The frequency cut-off value (ω_0) for the Grimme qRRHO approximation (default: `100.0` cm^{-1}).
- **`-no-H`**, **`--exclude-H`** If set, excludes Hydrogen atoms from RMSD and PCA distance matrix calculations.
- **`-cpu`** (int) Number of CPU cores to allocate for each QM calculation step (default: `1`).
- **`-calc`**, **`--calculator`** (str) Selects the external QM engine driver. Options: `"orca"` (default), `"gaussian"`.

Spectral Analysis (Graphing)

- **`--fwhm-vibro`** / **`--fwhm-electro`** Define the Full Width at Half Maximum (FWHM) for convolution.
  - Can be a single float (fixed value).
  - Can be a range `[min, max]` for auto-optimization against a reference spectrum.
- **`--shift-vibro`** / **`--shift-electro`** Define the spectral shift parameter.
  - **Vibronic (IR/VCD)**: Multiplicative scaling factor.
  - **Electronic (UV/ECD)**: Additive shift [eV].
- **`--invert`** Inverts the y-axis for chiral spectra (VCD/ECD).
- **`--definition`** (int) Resolution of the spectral grid, defined as 10^N points (default: `4`, i.e., 10,000 points).

Utilities

- **`-h`**, **`--help`** Shows the main help message.
- **`-h-p`**, **`--help-protocol`** Prints a JSON template for the protocol file.
- **`-h-t`**, **`--help-threshold`** Prints a JSON template for the threshold file.
- **`--disable-color`** Disables ANSI colored output in the terminal logs.

CHAPTER SEVEN

QUICKSTART

This tutorial guides you through a standard workflow: refining a conformational ensemble of a small molecule.

Prerequisites

Ensure you have the following files in your directory:

1. `ensemble.xyz`: The initial conformers (from crest/rdkit).
2. `protocol.json`: The calculation settings.

Step 1: Verify Setup

Check if your environment is ready:

```
enan_check
```

Step 2: define the protocol

Create a `protocol.json` file. You can either use the protocol wizard:

```
enan_protocol_wizard
```

Or use this standard template for: i) Single Point pruning, ii) Optimization + Frequency, and iii) re-evaluating the Electronic Energy:

```
{
    "0": {
        "functional": "B97-3c",
        "solv": {"solvent": "chcl3", "smd": true}
    },
    "1": {
        "functional": "r2scan-3c",
        "opt": true,
        "freq": true,
        "solv": {"solvent": "chcl3", "smd": false}
    },
    "2": {
        "functional": "wB97X-D4rev",
        "basis" : "def2-QZVPPD",
```

(continues on next page)

(continued from previous page)

```
        "solv": {"solvent": "chcl3", "smd": false}
    }
}
```

Step 3: Run the program

Launch the main executable:

```
ensemble_analyzer -e ensemble.xyz -p protocol.json -cpu 8
```

Step 4 Check Result

After completion, check the log file `output.out`

CHAPTER

EIGHT

EXAMPLE 1: MULTI-LEVEL ENSEMBLE REFINEMENT

This tutorial reproduces the **Ensemble Refinement** case study described in the paper. The goal is to refine a large ensemble of a flexible molecule generated by CREST (or any other conformational search tool existing).

Command

This command launches the refinement process using 8 CPU cores. The `-e` flag specifies the input ensemble, and `-p` points to our custom JSON protocol.

```
ensemble_analyzer -e ensemble.xyz -p protocol_refinement.json -cpu 8
```

Protocol

```
{
    "0": {
        "functional": "ExtOpt",
        "basis": "def2-mTZVP",
        "add_input": "\n%method ProgExt '/path/to/orca-external-tools/gxtb.py' end\n"
    },
    "1": {
        "functional": "r2SCAN-3c",
        "basis": "def2-mTZVPP",
        "opt": true,
        "freq": true,
        "skip_opt_fail": true
    },
    "2":{
        "functional": "wB97X-D4rev",
        "basis": "def2-QZVPP",
        "add_input": "\n! tightscf defgrid3\n",
        "read_orbitals": 1
    }
}
```

The protocol effectively reduces the number of structures by identifying duplicates and high-energy conformers.

The table below summarizes the retention rates achieved using this protocol on various flexible molecules, demonstrating the efficiency of the pruning strategy (results from **Table 1** of the manuscript).

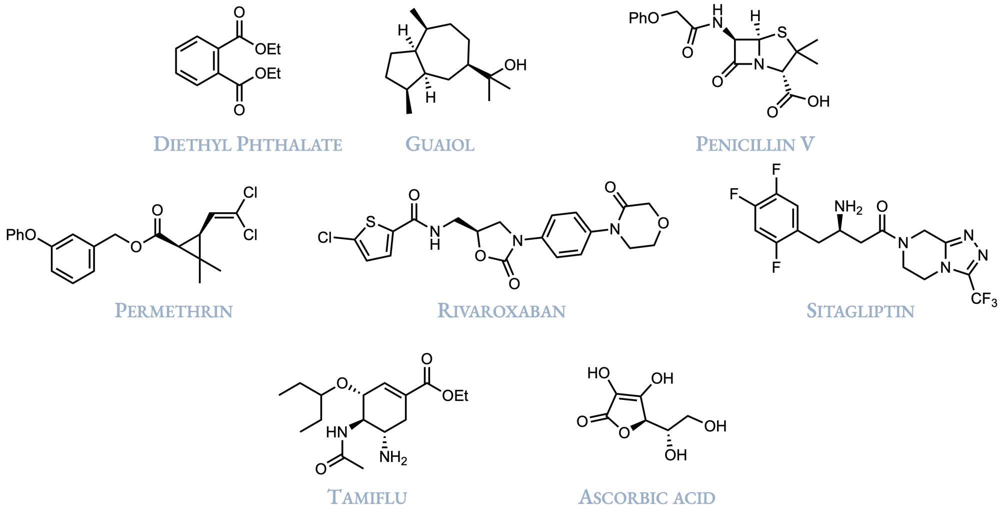

Molecule	Starting Ensemble	Protocol 1[a]	Protocol 2[b]	Protocol 3[c]	Final Retention Rate
**Diethyl Phthalate**	156	151	35	35	22 %
**Guaiol**	118	109	34	34	29 %
**Penicillin V**	136	71	12	12	9 %
**Permethrin**	304	304	101	101	33 %
**Rivaroxaban**	110	110	30	30	27 %
**Sitagliptin**	428	343	142	142	33 %
**Tamiflu**	836	527	100	99	12 %
**Ascorbic acid**	134	68	45	45	33 %

[a]: Single Point energy calculation at g-xTB level; [b]: Optimization and frequency calculation at r^{2}SCAN-3c level; [c]: Single Point energy evaluation at ωB97X-D4rev/def2-QZVPP level.

8.1 Clustering Impact Analysis

To evaluate the impact of the clustering discretization on ensemble-derived properties, three flexible molecules were selected as representative case studies: Vancomycin, Paclitaxel, and Prostaglandin PGF$_{2\alpha}$ (Figure S2).

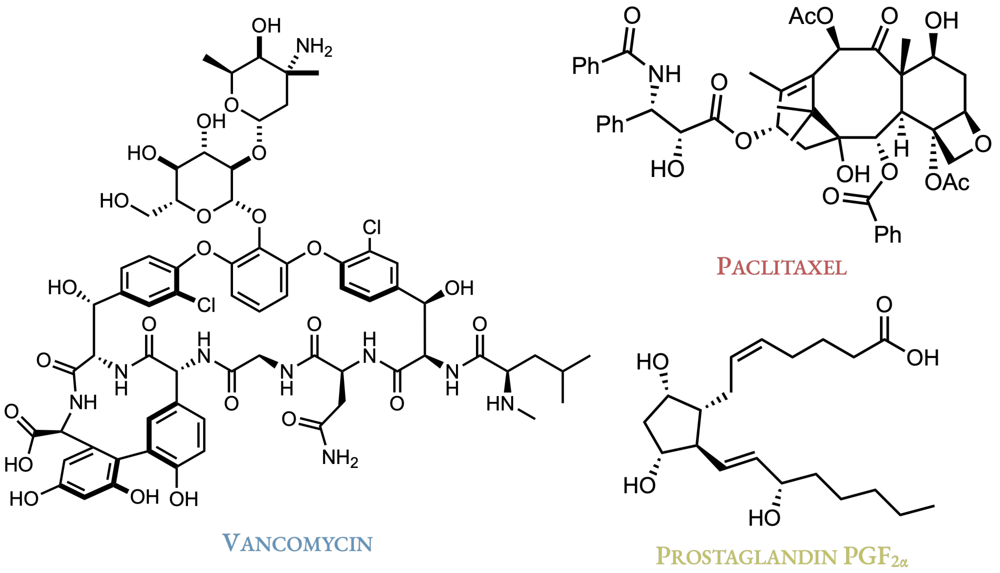

Figure S2: Flexible molecules used as case study for the clustering analysis.

Initial ensembles were generated using CREST, and the conformer energies were subsequently re-evaluated at g-xTB level directly within EnAn. Each ensemble was then clustered while systematically varying the number of resulting clusters. For each new clustered ensemble, the Boltzmann weighted average energy was calculated and compared against that obtained from the full ensemble.

The resulting trend, shown in Figure S3, reports the variation of the energy deviation ($\Delta E_t extav$) as a function of the percentage of conformers retained – thus illustrating the energy loss introduced by the progressively reducing of the number of clusters.

Ascorbic acid was also included; in this case, the values plotted were computed using Gibbs free energy, with electronic energy evaluated at the ωB97X-D4rev/def2-QZVPP level and the thermal correction calculated at r^{2}SCAN-3c level.

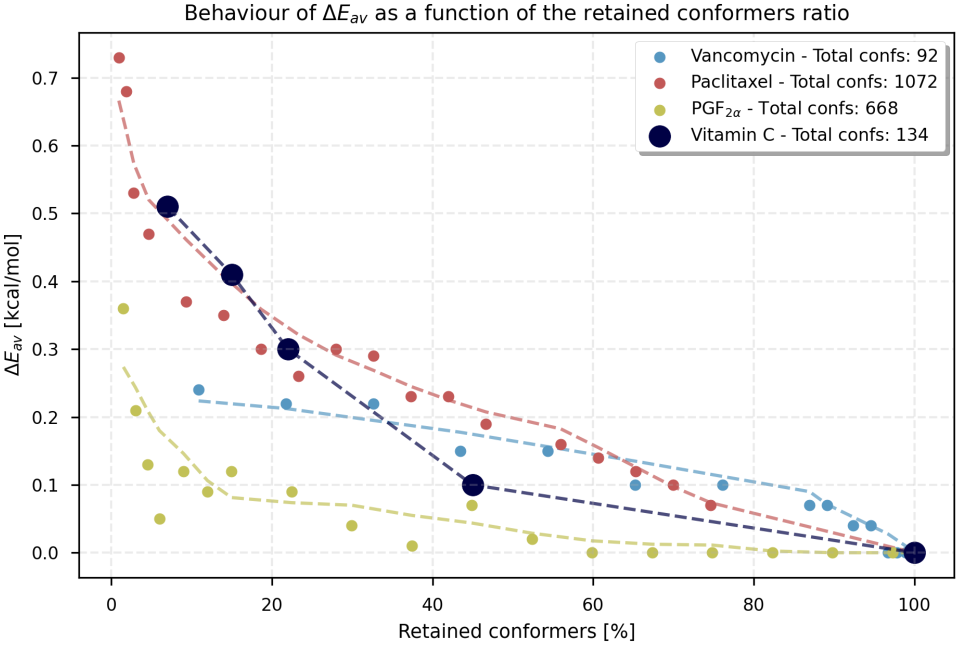

Figure S3: Variation of ΔE_{av} loss as a function of the conformer's retention.

As expected, reducing the number of conformers decreases accuracy, as reflected by the increase in $\Delta E_t extav$. Conversely, fewer conformers significantly reduce the wall time required for optimization and frequency calculation (see Figure S4).

Therefore, clustering the ensemble represents an excellent strategy for reducing the dimensionality of the conformational space and, consequently, the computational cost. However, it must be applied judiciously to balance efficiency and accuracy. To identify the optimal clustering granularity, EnAn employs an automated optimization strategy. The algorithm systematically evaluates all integer values of n (number of clusters) between 10% and 80% of the ensemble size, with a lower bound of two clusters. For each n value, K-means clustering is performed, and the corresponding silhouette score is calculated. The configuration yielding the highest silhouette score is selected as the optimal clustering solution.

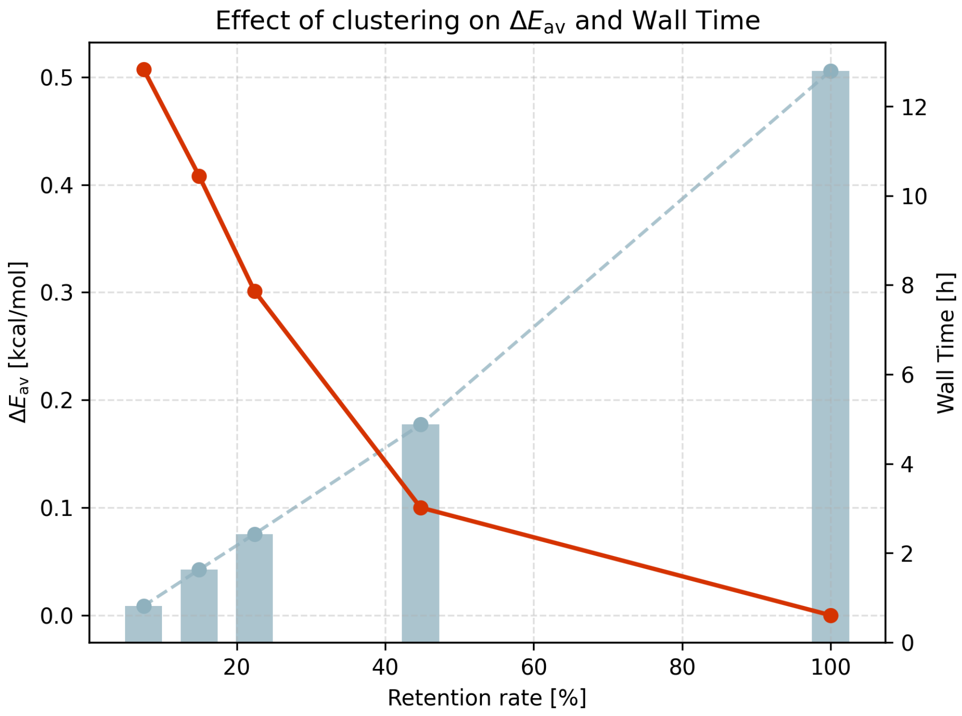

Figure S4: Trade-off function between accuracy (in red) and wall time (in blue) as a function of the retention rate. Performed on 44 Intel® Xeon® CPU cores.

CHAPTER

NINE

EXAMPLE 2: VCD AND ECD SPECTRA SIMULATION

In this example, we simulate the Vibrational Circular Dichroism (VCD) and the Electronic Circular Dichroism (ECD) spectra of a chiral molecule and automatically fit it against an experimental reference file (`vcd_ref.dat`, `ecd_ref.dat`).

Command

```
ensemble_analyzer -e ensemble.xyz -p protocol_vcd.json -cpu 44
```

Protocol

```
{
    "0": {
        "functional": "B97-3c",
        "basis": "def2-mTZVP"
    },
    "1": {
        "functional": "r2SCAN-3c",
        "basis": "def2-mTZVPP",
        "opt": true,
        "freq": true
    },
    "2": {
        "functional": "wB97X-D4rev",
        "basis": "def2-QZVPP"
    },
    "3": {
        "functional": "B3LYP D4",
        "basis": "def2-QZVPPD",
        "freq": true
    }
}
```

Result for the VCD spectra is shown in Figure S5

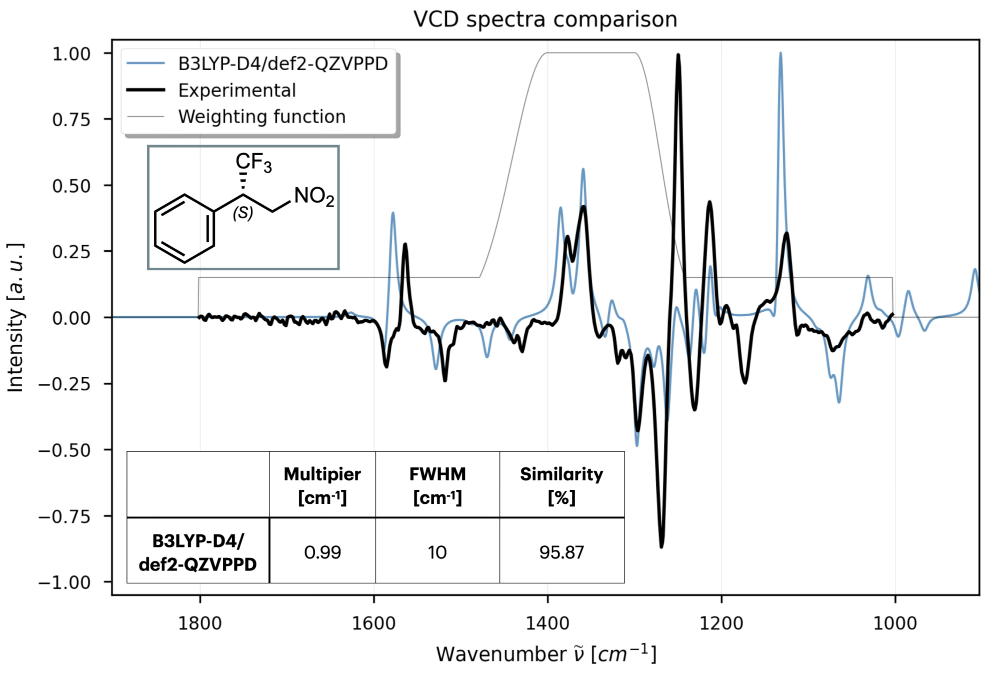

Figure S5: Convoluted VCD obtained with optimized parameters.

At the end of the calculation, two files per spectra will be stored: one `.pickle` file and one `.png` file. If you want to modify the automatically generated spectra, it is possible to use the terminal interface of the `enan_graph_editor` module, to customize the spectra.

To refine the interested area and the multiplier, it is possible to change the values stored in the `setting.json` file and use the CLI command `enan_regraph` to recompute the various spectra, without the need of relaunching the calculation.

The same workflow has been used to simulate the ECD spectra, but with the following ensemble and following protocol

Protocol

```
{
    "0": {
        "functional": "r2SCAN-3c",
        "basis": "def2-mTZVPP",
        "opt": true,
        "freq": true,
        "solvent":{
        "solvent": "acetonitrile",
        "smd": true
        }},
    "1":{
        "functional": "wb97X-D4",
        "basis": "def2-TZVPP",
        "solvent": {
            "solvent": "acetonitrile",
            "smd": true
        },
        "add_input":"%tddft nroots 40 TDA FALSE end"
```

(continues on next page)

(continued from previous page)

```
    },
    "2":{
        "functional": "CAM-B3LYP D4",
        "basis": "def2-TZVPP",
        "solvent": {
            "solvent": "acetonitrile",
            "smd": true
        },
        "add_input":"%tddft nroots 40 TDA false end",
        "read_orbitals": "1"
    }
}
```

At the end of the calculations, the final ECD comparison figure (FIgure S6) is saved (both as a `.pickle` and a `.png`).

Result for the VCD spectra is shonw in Figure S5

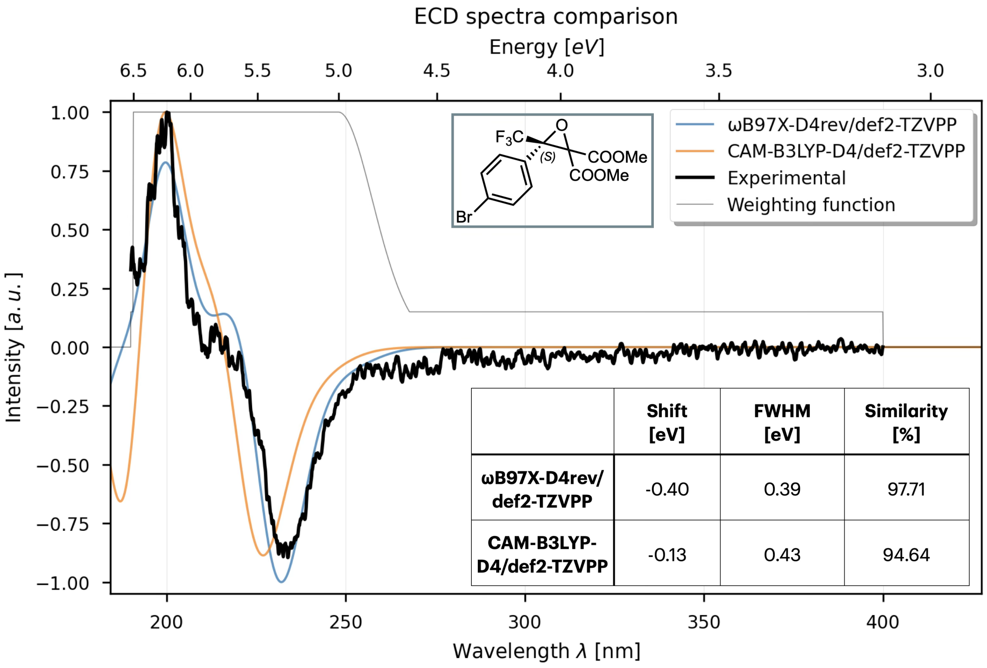

Figure S6: Convoluted ECD obtained with optimized parameters.

Showcasing of the Automated Spectral Fitting

To show the robustness and reliability of the automated spectral fitting procedure (shift and FWHM optimization via RMSD minimization), the algorithm was apllied across different spectral profiles.

UV-Vis Spectrum Fitting: Figure S7 demonstrates the baseline reliability of the algorithm by fitting a simulated UV-Vis spectrum (based on the model system from the ORCA 6.1 TD-DFT tutorial) against its experimental reference. The routine successfully identifies the optimal convolution parameters to match the experimental profile.

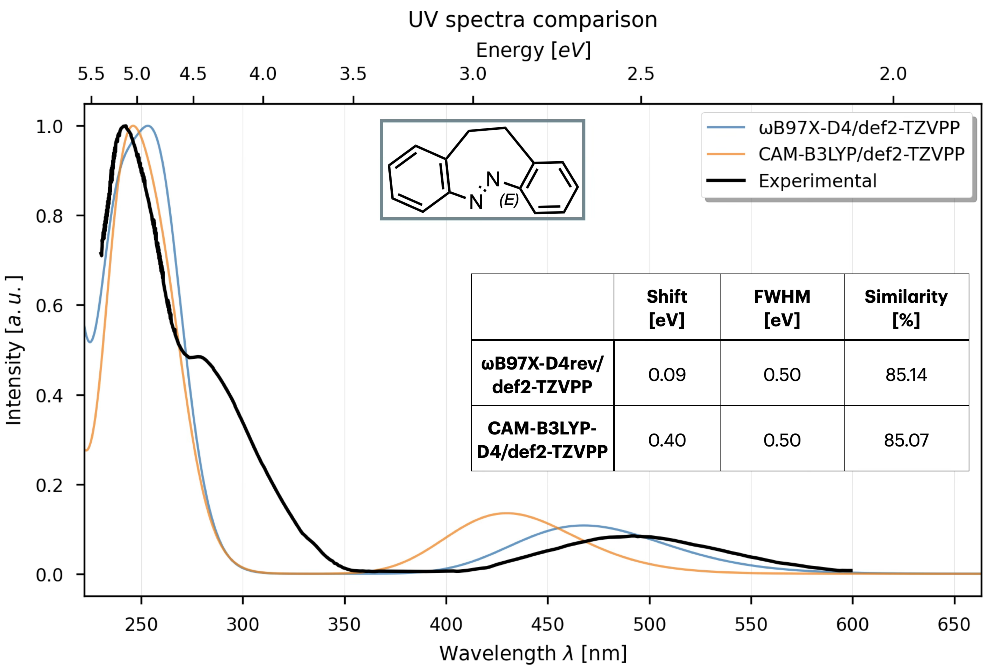

	Shift [eV]	FWHM [eV]	Similarity [%]
**ωB97X-D4rev/ def2-TZVPP**	0.09	0.50	85.14
**CAM-B3LYP-D4/def2-TZVPP**	0.40	0.50	85.07

Figure S7: Automated fitting of the simulated UV-Vis spectrum against the experimental reference.

Impact of the Weighting Function on ECD Spectra

Experimental spectra, particularly VCD and ECD, often contain complex transition regions or artifacts near the solvent cut-off limit. If the RMSD minimization is applied blindly across the entire recorded window, the optimizer seeks a global compromise, often skewing the shift or FWHM to minimize errors in less reliable regions. EnAn circumvents this through the user-definable weighting function ($w(x)$).

Figure S8 illustrates the fitting of an ECD spectrum evaluated with and without this function. Without the weighting function, the optimizer attempts to minimize the overall error across the complex transitions at lower wavelengths (< 240 nm), resulting in a visible, albeit small, misalignment of the primary diagnostic peak at ~260 nm. Conversely, by applying the weighting function to isolate this electronic transition, the optimizer correctly prioritizes this region, aligning the computed peak perfectly with the experimental data.

EnAn does not impose rigid fitting boundaries. The `enan_regraph` tool enables users to iteratively adjust the shift and FWHM boundaries, as well as the fitting window, post-calculation. This design choice provides both automation for standard cases and flexibility for challenging spectral profiles, allowing users to refine the fitting parameters without requiring expensive re-execution of the QM steps.

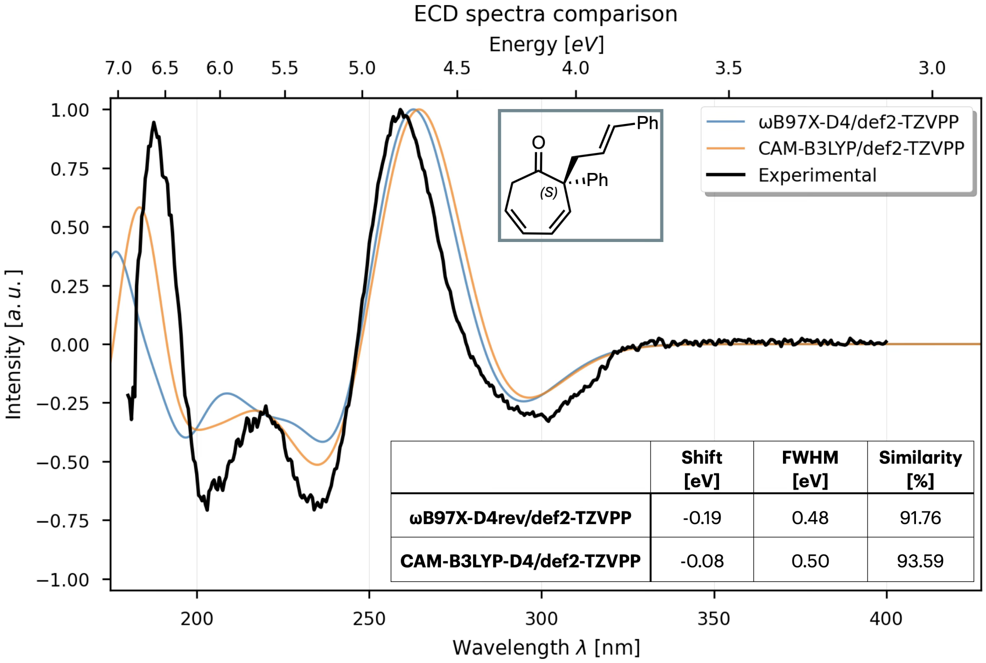

(a) ECD convoluted without using the weighting function

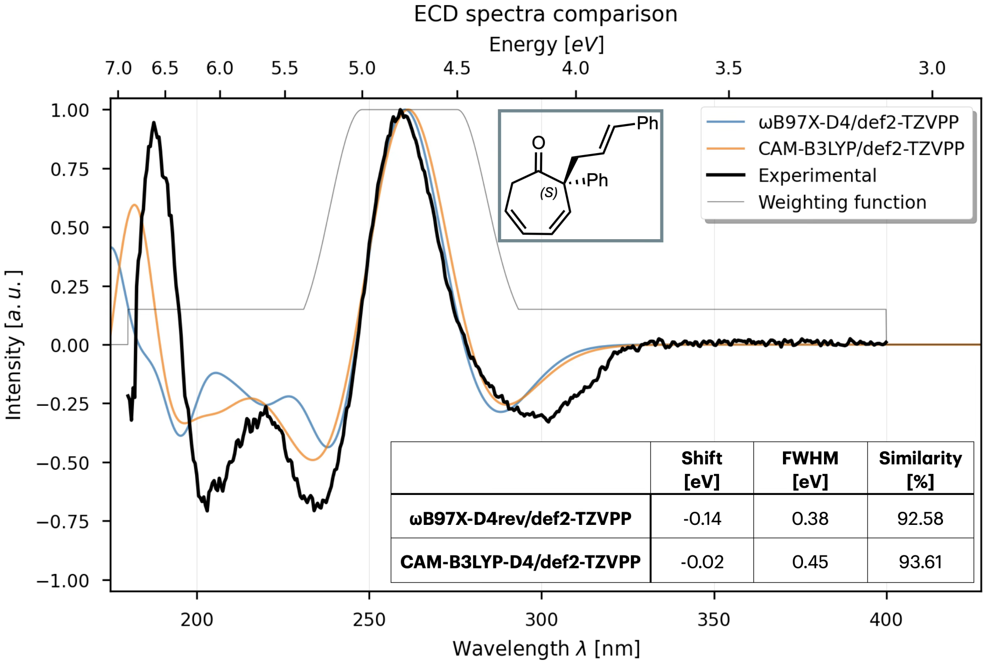

(b) ECD convoluted using the weighting function

Figure S8: Comparison of the automated ECD spectral fitting (a) without and (b) with the application of the weighting function.

CHAPTER TEN

EXAMPLE 3: SINGLET-TRIPLET ENERGY GAP (ΔE S→T)

This tutorial demonstrates how to calculate the **Singlet-Triplet energy gap** for an aminoborane derivative. The workflow involves manipulating the multiplicity settings across different steps to optimize Ground State (S_0), Excited Singlet (S_1), and Triplet (T_1) states.

Command

```
ensemble_analyzer -e ensemble_aminoborane.xyz -p protocol_st_gap.json -cpu 16
```

Protocol

```
{
    "0": {
        "functional": "r2SCAN-3c",
        "basis": "def2-mTZVPP",
        "solvent": {
            "solvent": "CHCl3",
            "smd": false
        },
        "mult": 1,
        "charge": 0,
        "opt": true,
        "freq": true,
        "no_prune": true,
        "comment": "Opt Freq S0"
    },
    "1": {
        "functional": "CAM-B3LYP D4",
        "basis": "def2-TZVPP",
        "solvent": {
            "solvent": "CHCl3",
            "smd": false
        },
        "mult": 1,
        "charge": 0,
        "no_prune": true,
        "comment": "SP S0"
    },
    "2": {
        "functional": "r2SCAN-3c",
        "basis": "def2-mTZVPP",
```

(continues on next page)

(continued from previous page)

```
        "solvent": {
            "solvent": "CHCl3",
            "smd": false
        },
        "calculator": "orca",
        "mult": 1,
        "charge": 0,
        "opt": true,
        "freq": true,
        "read_orbitals": "0",
        "add_input": "\n! DeltaSCF UKS FreezeAndRelease SCFCheckGrad VerySlowConv\n
↪%scf alphaconf 0,1 end",
        "no_prune": true,
        "comment": "Opt Freq S1"
    },
    "4": {
        "functional": "CAM-B3LYP D4",
        "basis": "def2-TZVPP",
        "solvent": {
            "solvent": "CHCl3",
            "smd": false
        },
        "calculator": "orca",
        "mult": 1,
        "charge": 0,
        "opt": false,
        "freq": false,
        "no_prune": true,
        "comment": "Prepare SP S1"
    },
    "5": {
        "functional": "CAM-B3LYP D4",
        "basis": "def2-TZVPP",
        "solvent": {
            "solvent": "CHCl3",
            "smd": false
        },
        "mult": 1,
        "charge": 0,
        "read_orbitals": "4",
        "add_input": "\n! DeltaSCF UKS FreezeAndRelease SCFCheckGrad\n %scf alphaconf␣
↪0,1 end %pal nprocs_group 4 end",
        "no_prune": true,
        "comment": "SP S1"
    },
    "6": {
        "functional": "r2SCAN-3c",
        "basis": "def2-mTZVPP",
        "solvent": {
            "solvent": "CHCl3",
            "smd": false
        },
```

(continues on next page)

(continued from previous page)

```
        "mult": 3,
        "charge": 0,
        "opt": true,
        "freq": true,
        "no_prune": true,
        "comment": "Opt Freq T1"
    },
    "7": {
        "functional": "CAM-B3LYP D4",
        "basis": "def2-TZVPP",
        "solvent": {
            "solvent": "CHCl3",
            "smd": false
        },
        "calculator": "orca",
        "mult": 3,
        "charge": 0,
        "no_prune": true,
        "comment": "SP T1"
    }
}
```

The workflow results in the energy values summarized in the diagram in Figure S9.

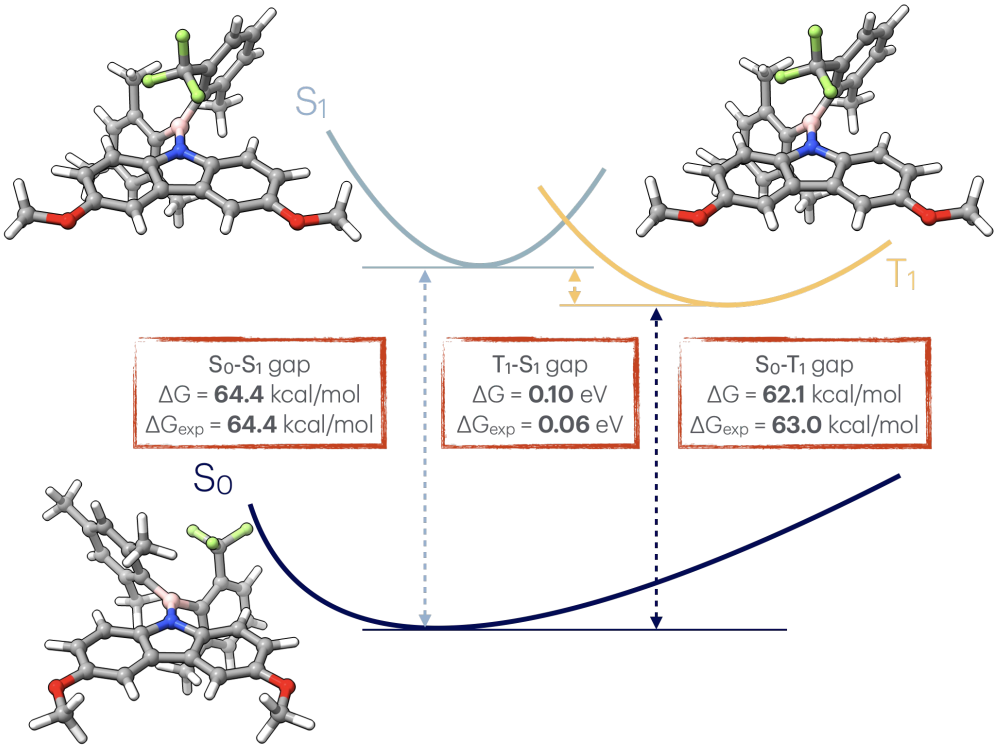

Figure S9: Simplified Jablonski diagram

The final results can be schematically obtained using the CLI command `enan_get_energy`, which can also perform

some basic algebraic calculation between the average energies of the various protocols:

```
enan_get_energy --sub 5 1 \ # S0-S1 adiabatic gap
    --sub 7 1 \ # S0-T1 adiabatic gap
    --sub 5 7  # T1-S1 adiabatic gap
```

Gap	Energy	Experimental Value
S_0-S_1 gap	ΔG = 64.4 kcal/mol	ΔG = 64.4 kcal/mol
S_0-T_1 gap	ΔG = 62.1 kcal/mol	ΔG = 63.0 kcal/mol
T_1-S_1 gap	ΔG = 0.10 eV	ΔG = 0.06 eV

CHAPTER

ELEVEN

EXAMPLE 4: TRANSITION STATE ENSEMBLE REFINEMENT

Ensemble Analyzer can also handle **Transition State (TS)** refinement. This example reproduces the refinement of a TS ensemble for a model Diels-Alder reaction (673 conformers generated via GOAT).

The key features used here are: i) **clustering** to reduce the ensemble size, ii) **constrained optimization** to preserve the reaction center before the final TS optimization, and iii) the actual saddle point optimization via triggering the OptTS keyword.

Command

```
ensemble_analyzer -e ts_ensemble.xyz -p protocol_ts.json -cpu 44
```

Protocol

```
{
    "0": {
        "functional": "xTB",
        "cluster": 30,
            "comment": "Clustering"
   },
    "1": {
        "functional": "r2SCAN-3c",
        "basis": "def2-mTZVPP",
        "opt": true,
        "comment": "Constrained Optimization",
        "constrains": [0,1,2,3]
    },
    "2": {
        "functional": "r2SCAN-3c",
        "basis": "def2-mTZVPP",
            "freq": true,
        "add_input": "\n! OptTS \n%geom calc_hess true end\n",
        "read_orbitals": "1",
        "comment": "Berny"
    },
    "3": {
        "functional": "wB97X-3c",
        "comment": "SP"
    }
}
```

Note: The atom indices in the constraints block must be adjusted to match the reacting atoms in your specific xyz file. Numbering starts at 0.

The low-lying conformer after the refinement of the ensemble has an energy 4.84 kcal/mol **lower** than the original TS obtained from the Nudged Elastic Band. The geometry and the reaction is shown in Figure S10.

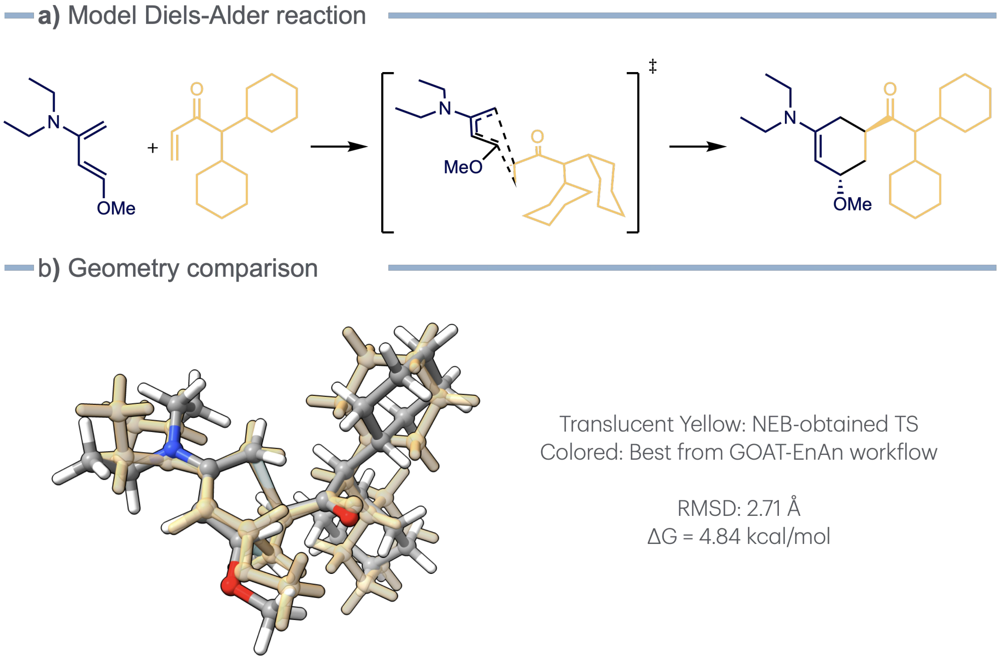

Figure S10: Summary of the TS conformational search.
